# A Tangentially Sensitive Tactile Sensor Reveals the Stick‐Slip Mechanism and Enhances Robotic Tactile Sensing

**DOI:** 10.1002/advs.202517884

**Published:** 2025-11-20

**Authors:** Jinghui Wang, Xiaoyu Liu, Yujiao Du, Yepu Chen, Jieyi Guo, Linyuan Fan, Min Tang, Xiaofeng Qiao, Yuanjie Zhu, Zhiyang Zhang, Chaojie Dong, Lizhen Wang, Yubo Fan

**Affiliations:** ^1^ Key Laboratory of Biomechanics and Mechanobiology (Beihang University) Ministry of Education Key Laboratory of Innovation and Transformation of Advanced Medical Devices Ministry of Industry and Information Technology National Medical Innovation Platform for Industry‐Education Integration in Advanced Medical Devices (Interdiscipline of Medicine and Engineering) School of Biological Science and Medical Engineering Hangzhou International Innovation Institute Beihang University Beijing 100191 China

**Keywords:** aerogel, pacinian corpuscle, robotic perception, stick‐slip, tactile sensor

## Abstract

The mechanism of human tactile perception offers inspiration for developing a high‐performance artificial sensory system. Pacinian corpuscles (PCs) allowing high‐frequency tangential sensitivity that endows humans with the ability to perceive multidimensional tactile stimuli. However, replicating similar high‐frequency and tangential perception as that of PCs in artificial systems remains a significant challenge. Here, an artificial PC (APC) from ultralight aerogel with a multilayer‐stacked and anisotropic structure analogous to that of PC via a multi‐directional freeze‐drying method is reported. The APC sensor with radial‐axial anisotropy exhibits tangential sensitivity (1022 kPa^−1^), detects multidimensional tactile stimuli, and identifies stick‐slip states with high precision. By mimicking PC clusters, the APC sensor system achieves spatiotemporal sensing capabilities for discriminating frictional patterns (static, sliding, or rolling) and tracking slip. A robot equipped with the APC sensory system possesses enhanced tactile perception for diverse tactile events in active (100% accuracy) and passive (98.18% accuracy) interaction with humans. It is found that high‐frequency components are crucial for stick‐slip detection, providing insights into the underlying tangential tactile sensing mechanisms of PCs. The work exemplifies a bidirectional enhancement of understanding between technological innovation and biological mechanisms.

## Graphical abstract

An ultralight, multilayer anisotropic tactile sensor—an artificial Pacinian corpuscle—exhibits ultrahigh tangential sensitivity (1022 kPa^−1^) and spatiotemporal sensing. It discriminates static, sliding, and rolling contacts, detects incipient stick–slip via high‑frequency signatures, and enhances robotic touch (100%/98.18% accuracy for active/passive interaction), offering insight into the tangential sensing mechanisms of Pacinian corpuscles.

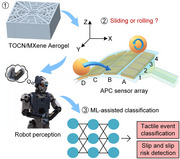

## 1 Introduction

Biomimetic strategies and technologies have led to the development of unconventional artificial tactile sensory systems (ATSSs) that partially replicate the structural and functional characteristics of the human tactile perception system (HTPS), including fingerprint‐like structures, hair‐follicle‐inspired configurations, and mechanically gated ion channel mechanisms.^[^ ^1^, ^2^, ^3^, ^4^, ^5^, ^6^, ^7^, ^8^ ^]^ These ATSSs have exhibited outstanding performances and significant potential for the next generation of human–robot interaction applications, such as texture recognition, shape, and hardness identification.^[^ ^9^, ^10^, ^11^, ^12^, ^13^, ^14^ ^]^ Despite extensive advances, current advancements in ATSSs are still largely limited to the detection of the normal component of pressures.^[^ ^15^ ^]^ Replicating the tangential perception capability of the HTPS remains a great challenge. This limitation constrains the performance of ATSSs in slip detection and moving‐object identification, which are essential for intelligent recognition, hazard response, and interactive feedback in robotics.^[^ ^16^ ^]^

**Figure 1:**
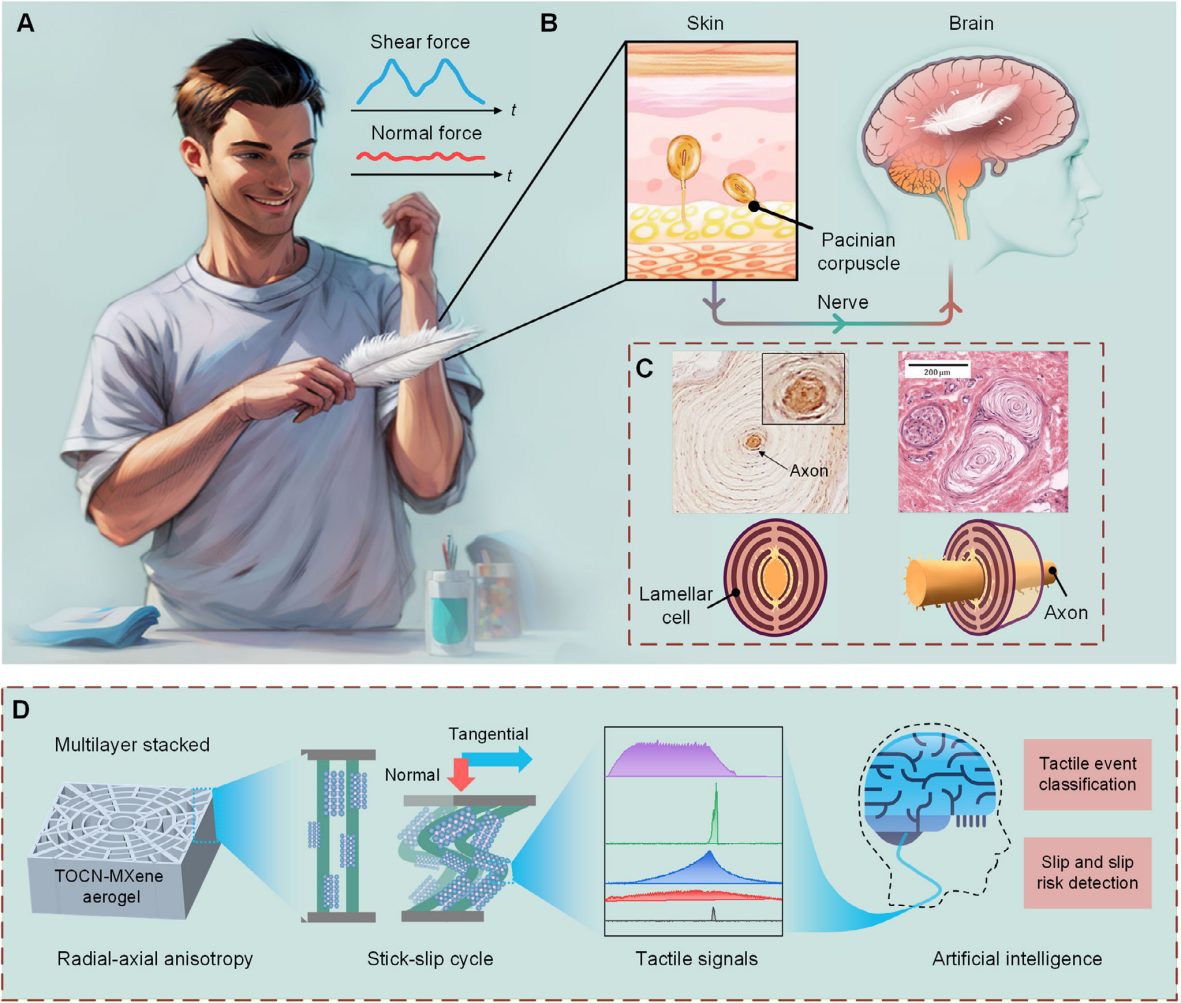
Structure and function of PCs and APC. A) Schematic illustration of human tactile sensing under feather sliding. Human figure generated using ComfyUI (v0.3.59) with Stable Diffusion 3, rpg_V4 (accessed October 2024) based on the authors’ hand‐drawn sketch. The content was directed and reviewed by the authors. B) Magnified schematic illustrating PCs residing in dermis, being sensitive to vibrations, and serving to a mechanical‐to‐electrical transduction. C) Top left: Section of PCs shows localization of Piezo2 in human palmar aponeurosis. The inset shows the axon at the core. Reproduced with permission.^[^ ^23^ ^]^ 2021, Wiley. Top right: A cluster of PCs in a human skin section. Reproduced with permission.^[^ ^30^ ^]^ 2018, Springer. Bottom: Schematic illustration of the inner region of a PC in front and lateral view. D) The tactile sensor of this study, for which the TOCN‐MXene aerogel‐based APC enables tactile‐event classification and slip/slip‐risk detection.

As a result of evolutionary adaptation, humans possess the ability to detect tangential stick‐slip and reciprocating sliding motions that generate high‐frequency components (**Figure** **1**A), in which the tangential force components exhibit larger variations than the normal component. In HTPS, the detection of tangential vibrations relies on the collaboration of Meissner and Pacinian corpuscles (PCs).^[^ ^17^, ^18^ ^]^ PCs are widely reported to be highly sensitive to high‐frequency vibrations above 40–50 Hz, most sensitive ≈200 Hz.^[^ ^19^ ^]^ Moreover, as the deepest cutaneous mechanoreceptors, PCs are located in the dermal tissue of hairy and glabrous skin, where they transduce multidimensional mechanical forces into electrical signals that propagate to the central nervous system via afferent axons (Figure 1B).^[^ ^17^, ^20^ ^]^ However, it is poorly known how the tangential acting force on the skin surface generates high‐frequency components and is transduced by PCs.

The biomechanical characteristics and perceptual mechanisms of PCs are widely believed to originate from their unique structure.^[^ ^20^, ^21^, ^22^ ^]^ In vitro cellular studies have shown that while the naked axon of the PC retains basic mechanoreceptive properties, it loses its rapid adaptation and off‐response properties,^[^ ^22^ ^]^ suggesting that the specialized structure of PCs plays a critical role in shaping their neural responses. Within the corpuscle, the axon is enveloped by the multilayered, stacked lamellae (Figure 1C).^[^ ^23^, ^24^, ^25^ ^]^ Also, the multilayered stacked lamellae that extend along the axial direction, leading to axial‐radial anisotropy.^[^ ^25^, ^26^, ^27^ ^]^ In addition, multiple PCs often form clusters functionally analogous to a sensor array.^[^ ^24^, ^26^ ^]^ Biomimetic PCs have been reported, including a midspan‐loaded beam that emulates a single PC‐like layer,^[^ ^28^ ^]^ and microphones.^[^ ^27^ ^]^ However, these designs did not focus on mimicking the overall structure of PC. In addition, biomimetic PCs are commonly employed to detect normal vibrations,^[^ ^29^ ^]^ whereas frequency perception induced by tangential motion is equally important for enhancing the performance of ATSSs. Nevertheless, this aspect has seldom been discussed.

Here, we reported an ATSS fabricated from an ultralight MXene/2,2,6,6‐tetramethylpiperidin‐1‐oxyl (TEMPO)‐mediated oxidized cellulose nanofiber (TOCN) aerogel (APC) with a multilayer stacked and anisotropic structure (Figure 1D). Developed via a multi‐directional freezing‐drying method, the APC exhibits radial‐axial anisotropy and high‐frequency sensitivity, allowing it to detect tangential stimuli and stick‐slip behavior with high precision. After optimization, the APC achieves an ultrahigh tangential sensitivity (1022 kPa^−1^), and fast response‐relaxation time (53.4 and 47.8 ms). By mimicking PC clusters, the APC array system (4 × 4) can achieve multidimensional sensing capabilities with spatiotemporal information, such as contact contour, frictional patterns (static, sliding, or rolling), vibration, and slip tracking. We mounted the system on a robotic arm, interacting with humans to examine its practical applications. The results indicate that the robot is able to identify multiple tactile events in active and passive human‐robot interaction with high accuracy (100% and 98.18%). Moreover, we found that high‐frequency components from tactile signals are crucial for slip detection and their feature recognition (e.g., occurrence time, velocity, direction). Our biomimetic work exemplifies a bidirectional enhancement between technological innovation and biological insights.

## 2 Results and Discussion

### 2.1 Fabrication and Sensing Mechanism of the APC

**Figure 2:**
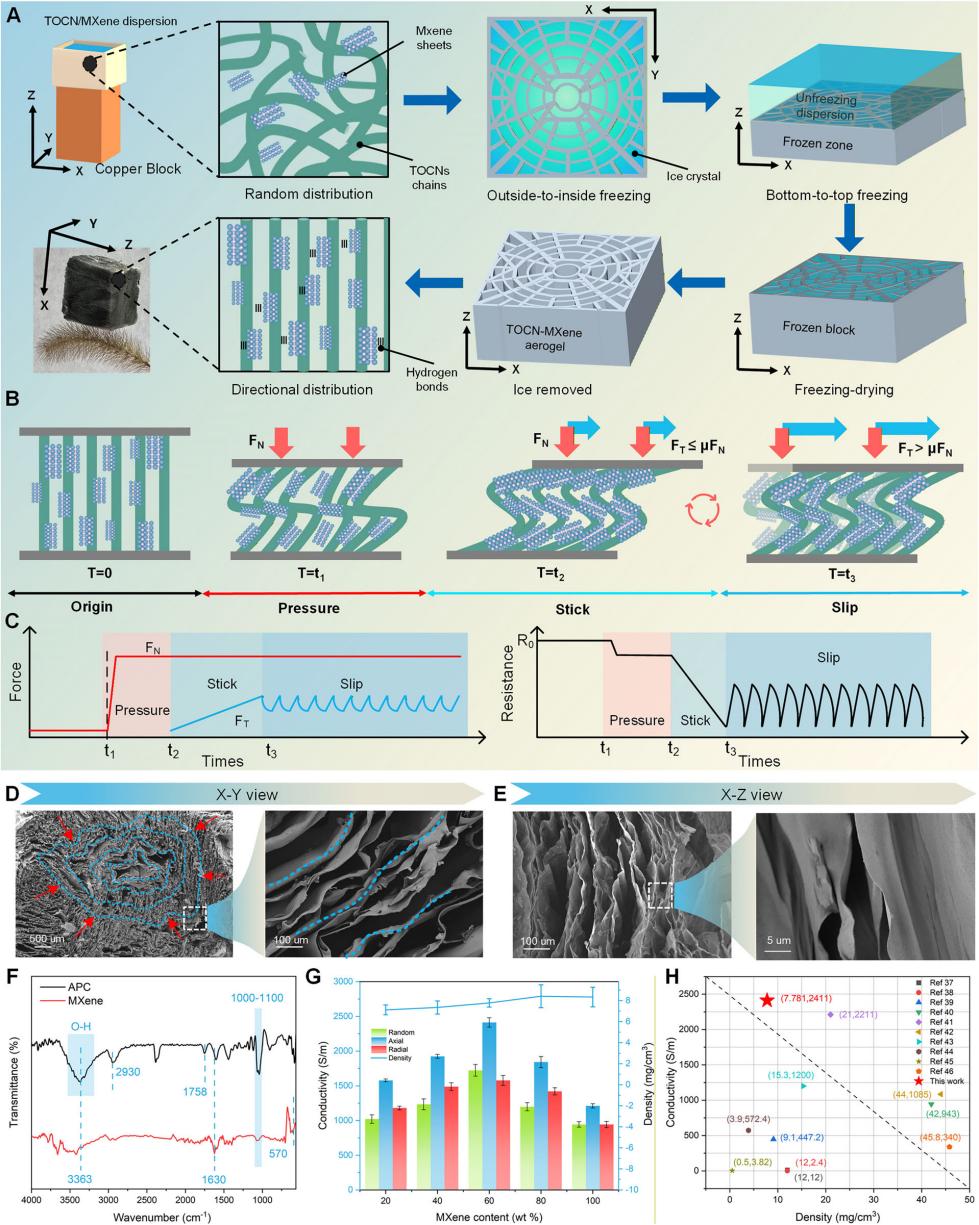
Fabrication, mechanism, and characterization of the APC. A) Schematic illustration of the fabrication process of the APC. The APC placed on a *setaria viridis* demonstrates the ultralight nature. B) Deformation process of the biomimetic lamellar structure of the APC under external force, referring to load‐free (T=0), normal force (T = *t*_1_), tangential force with stick state (T = *t*_2_), and slip state (T = *t*_3_). C) Change of normal force and tangential forces in different deformation processes (left), and the corresponding resistance response of the APC (right). The SEM images of the APC in (D) X‐Y plane (radial direction) and E) Y‐Z plane (axial direction). F) FT‐IR spectra of the MXene and the APC. G) Axial and radial conductivities of the APC and control samples with different MXene contents. The line plot represents the density of the APC. Error bars represent the SDs of the density in 5 measurements. H) Comparative analysis of the conductivity and density between our APC and aerogels reported in previous literature.

In human skin, the sensitivity of PCs to high‐frequency vibrations is widely ascribed to their specialized lamellar structure.^[^ ^20^ ^]^ The structure of PCs can be conceptualized as radially nested lamellae extending along the axial direction, forming an axial‐radial anisotropy. Here, we endeavored to develop an APC that replicates key characteristics of PCs structure. Specifically, we employed an innovative multidirectional freeze‐drying technology to fabricate the APC from an aqueous composite dispersion containing titanium carbide MXene nanosheets (Ti_3_C_2_T_x_) and TEMPO‐oxidized cellulose nanofibrils (TOCNs) (**Figure** **2**A). The MXene sheets (Figure S1, Supporting information) were obtained by etching and exfoliating Ti_3_AlC_2_ MAX phase precursors using LiF/HCl solution to selectively remove the Al layers.^[^ ^31^, ^32^ ^]^ The 1 wt.% aqueous suspension of TOCNs was prepared by fully disintegrating cellulose bundles into cellulose nanofibers through a 2,2,6,6‐tetramethylpiperidin‐1‐oxyl (TEMPO)‐mediated oxidation process (Figure S2, Supporting Information).^[^ ^33^, ^34^ ^]^ The MXene‐TOCNs dispersion was then subjected to a custom‐designed mold for multi‐directional freezing‐drying. TOCNs serve to suppress the restacking of MXene sheets by modulating interfacial interactions through their abundant surface functional groups.^[^ ^35^ ^]^ During the freezing process, a copper block (length 21 mm, width 21 mm, height 100 mm) immersed in liquid nitrogen formed a bottom‐to‐top temperature gradient, from −10 °C (mold bottom) to 26 °C (room temperature), while ice packs placed around the perimeter induced an additional outside‐to‐inside gradient, from 0 °C (mold periphery) to 26 °C (room temperature).^[^ ^36^ ^]^ Phase separation was triggered upon reaching the critical temperature (0 °C), followed by the directional growth of ice crystals along the temperature gradients. The FEA results also confirm the temperature gradients (Figure S3, Supporting information).^[^ ^37^ ^]^ Simultaneously, the hydrogen bond‐crosslinked MXene and TOCN were expelled to align alongside the grown ice crystals due to the volume exclusion effect. After completely freezing, an anisotropic ice crystal structure was formed, characterized by multilayered stacking in the X‐Y plane (radial direction) and unidirectional growth along the *z*‐axis (axial direction). The MXene and TOCN components were largely guided by this spatial configuration, aligning accordingly within the frozen block. Finally, PCs inspired MXene/TOCN aerogels (APC) with the multilayered stacked structure were obtained after freeze‐drying to remove ice templates (Figure S4, Supporting information). The 1D TOCNs at high concentrations (1 wt.%) acted as “threads” that interwove and anchored the MXene sheets within the fibrous network. The configuration of MXene sheets is largely dominated by the alignment and continuity of the 1D nanofibrils, thereby maintaining a stable and continuous framework. An as‐prepared APC exhibits ultralight weight and high porosity, allowing a volume of 8 cm^3^ to be supported by the tip of a *Setaria viridis* (Figure 2A). Moreover, the shape and size of the APC can be tuned by altering mold designs (Figure S5, Supporting information).

The multilayered, stacked, and anisotropic structure endows the APC with multidimensional sensing capabilities (Figure 2B). A normal force (*F_N_*) causes APC buckling in axial, resulting in height reduction and radial expansion (*T* = t_1_). Microscopically, an increase in the contact area between compressed MXene sheets enhances conductive pathways and decreases the resistance (*R*) (Figure 2C). When an additional tangential force (*F_T_*) is applied to the APC (*T* = t_2_), the contact remains in “stick” (or static friction) state as long as F_t_ ≤ µF_n_, where µ is the coefficient of friction. The static friction induces unidirectional buckling and folding of the well‐aligned lamellae at the microscopic scale, resulting in the storage of potential energy from the elastic deformation. In terms of electrical properties, a significant enhancement in conductive pathways within the APC leads to a sharper decline in *R*, with a slope steeper than that recorded at *T* = t_1_. When *F*_t_ > µ*F_N_* (*T* = t_3_), the contact fails to maintain “stick” state and exhibits “stick‐slip” behavior. The first “slip” releases the stored potential energy in the stick state rapidly, leading to a fast deformation recovery of compressed MXene sheets. The resistance of the APC increases correspondingly. After the first “slip,” the system then reaches a new quasi‐steady state, characterized by alternating cycles of sticking (static friction) and slipping (kinetic friction), accompanied by repeated cycles of compression and relaxation within the multilayered lamellar space due to enhanced interlayer conformity. Thus, the resistance of the APC exhibits high‐frequency oscillations, with the frequency strongly related to the motion velocity. These findings demonstrate that the multilayered and anisotropic structure not only enhances tangential sensitivity relative to normal force but also enables the APC to precisely distinguish between static and kinetic friction based on its high‐frequency electrical response.

### 2.2 Biomimetic Structure Allowing High Conductivity and Ultralight for the APC

We modulated i) the freezing process and ii) the mass fractions of MXene to tailor the structure and performance of the APC. Control aerogels, referred to as “random” aerogels, were prepared by placing the samples in a conventional freezer at −4 °C. In addition, aerogels with various MXene mass fractions (20%, 40%, 60%, 80%, and 100%) were prepared as detailed in the Experimental Section section and Table S1 (Supporting Information).

Scanning electron microscopy (SEM) images (X‐Y view) show that the APC consists of a multilayered stacked MXene‐TOCN network with a symmetrically disordered porous core in the center (Figure 2D). However, magnified views reveal that the multilayered stacking is approximately parallel in the peripheral region, with a lamellar thickness of ≈0.5 µm and an interlamellar spacing of ≈100 µm (Figure S6, Supporting information). The central disorder and peripheral well‐aligned arrangement originate from the outside‐to‐inside temperature gradient. The solution in the peripheral region freezes earlier than that in the central region, resulting in structural heterogeneity along the radial direction. In the X‐Z view, SEM images reveal unidirectional ice crystal growth, with the TOCN–MXene phase expelled into the interstitial regions to form lamellar walls (Figure 2E). Magnified views show smooth and crack‐free surfaces of the lamellar walls, which can be attributed to the thermodynamically driven alignment of TOCNs along the bottom‐to‐top freezing direction. This alignment effectively encapsulates MXene within the fibrous matrix, resulting in a composite structure that combines electrical conductivity with mechanical strength. In contrast, the random group fabricated by the freezer exhibits the irregular porous microstructure (Figure S7, Supporting information). Additionally, the presence of Ti, O, and C elements in the EDS mapping further confirms the MXene and TOCNs skeleton (Figure S8, Supporting information). Ti is uniformly distributed, while C and O exhibit layered distributions, indicating that MXene is well‐dispersed within the TOCN scaffold without significant aggregation (Figure S9, Supporting Information). Fourier‐transform infrared spectroscopy (FT‐IR) analysis was further employed to examine the interactions between MXene and TOCNs (Figure 2F). In the spectrum of the APC, characteristic peaks from MXene and TOCNs are retained, including the ─OH stretching (≈3420 cm^−1^), the C–F stretching (1000–1100 cm^−1^), and Ti─O and Ti─C vibrations (500–800 cm^−1^) from MXene, and peaks at ≈1758 cm^−1^ (C = O stretching) and ≈2930 cm^−1^ from TOCNs. This demonstrates the integration of MXene within the TOCNs network. Notably, the broad O─H band shows a redshift and increased intensity, suggesting strong hydrogen bonding interactions between the MXene and TOCNs.

To achieve optimal sensitivity to external force stimuli, the APC should be designed with an MXene content slightly exceeding the electrical percolation threshold.^[^ ^34^ ^]^ We measured the conductivity of APCs with different MXene contents to determine an optimal composition (Figure 2G). When the MXene content is below 60%, the conductivity increases rapidly. However, beyond the percolation threshold (≈60 wt.%), further increases in MXene content led to a decrease in conductivity, attributable to flake restacking that reduces effective inter‐flake junctions and raises contact resistance. Therefore, we selected 60 wt.% MXene to fabricate the APC sensors. Finally, the APC exhibits higher conductivity (2411 S m^−1^) along the axial than that (1877 S m^−1^) along the radial direction. Such anisotropy arises from the bottom‐up alignment of TOCNs, which guides the orientation of MXene sheets. In contrast, MXene sheets in the radial plane are distributed in stacked lamellae, which limits effective electrical connection. Additionally, “random” aerogel as a control group shows uniform conductivity in all directions, lacking the anisotropic characteristics. Also, the APC exhibits remarkably ultralight density of 7.781 mg cm^−3^, because surface functional groups of MXene generate strong interactions with TOCNs to form numerous voids to serve as pressure‐absorbing domains. Compared to previously reported conductive aerogels,^[^ ^38^, ^39^, ^40^, ^41^, ^42^, ^43^, ^44^, ^45^, ^46^, ^47^ ^]^ our APC exhibits superior conductivity and ultralow density (Figure 2H), thereby making it a promising candidate for slight tactile sensing.

### 2.3 Evaluation of Mechanical and Piezoresistive Properties of the APC

**Figure 3:**
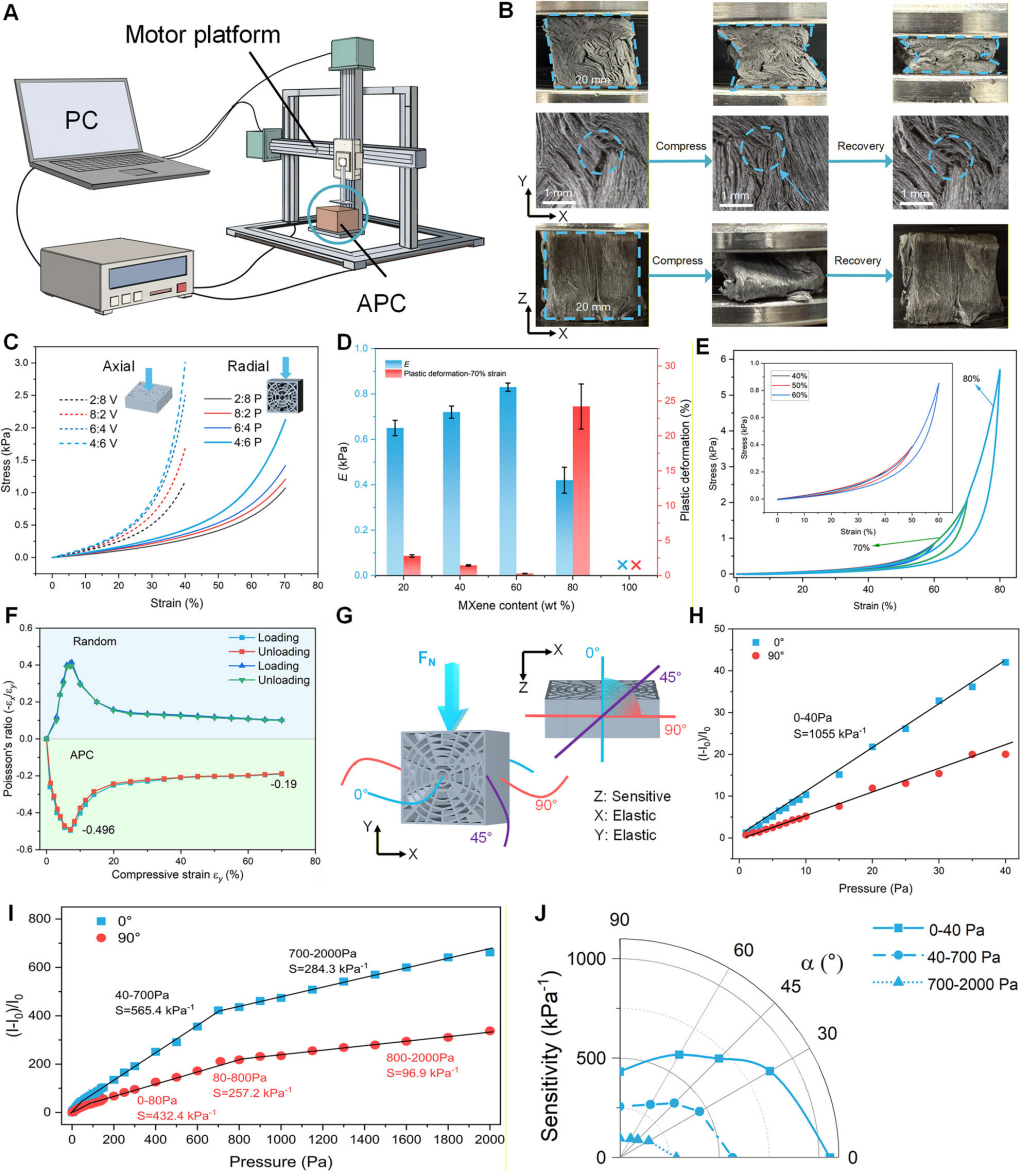
Mechanical and piezoresistive properties of the APC. A) Schematic of the custom‐built testing platform for measuring the mechanical and piezoresistive properties of the APC. B) Deformation images of the APC under radial compression (top), metallurgical microscopy images under radial compression and recovery (middle, scale bars: 1 mm), and deformation images under axial compression and recovery (bottom). C) Stress‐strain curves of the APC with different MXene contents (20%, 40%, 60%, and 80%) under axial and radial compression. D) The elastic modulus (*E*) and plastic deformation under the axial compression (strain 70%). Error bars represent the SDs of the *E* and plastic deformation in 5 measurements. E) Hysteresis curves of the APC under cyclic loading with strains up to 80%. Inset: curves under cyclic loading with strains up to 60%. F) Changing Poisson's ratio under loading‐unloading cycle up to strain of 70% for the APC and control samples. G) Schematic diagram of the directional sensitivity of the APC. Under radial compression along the *y*‐axis, the sensitivity is maximal at 0° (*z*‐axis) and decreases as the measurement orientation rotates toward 90° (*x*‐axis). H) Relative current changes (*(I‐I_0_)/I_0_*) and force sensitivity of the APC under pressure 0‐40 Pa and I) across a full range between 0 and 2000 Pa along the direction 0° and 90°, respectively. J) Polar plot of sensitivity under different pressures and directional angles (up to 90°).

The inherent characteristics of the APC play a crucial role in determining its sensing performance. To validate this, we investigated how the mechanical and piezoresistive properties of the APC contribute to tactile sensing. Here, we developed a mechanical testing platform with a mobile indenter, capable of applying normal and tangential forces on the sample (**Figure** **3**A). The force sensor was mounted on the indenter to detect the contact forces and decouple the normal and tangential components (Supporting Text). During the measurement, the APC sample was fixed on a horizontal plane. By controlling the vertical position of the indenter (*z*‐axis), a specific normal force could be applied to the APC. Additionally, precise control of the indenter's displacement and velocity along the *x*‐axis enabled the application of tangential forces.

The APC exhibits high stiffness along the axial direction and good elasticity along the radial direction (Figure 3B). When subjected to uniaxial compression in the radial direction, the digital image of APC (top) exhibits a negative radial strain, contrasting with the radial expansion observed in most materials under compression. Metallurgical microscopy images (middle, 10×magnification) show the contact and separation between stacked layers in the APC in compression and release, which may underlie robust piezoresistive behavior. In addition, the axial compress leading APC buckling, resulting in height reduction and radial expansion. The anisotropic mechanical behavior was observed when the APCs were subjected to normal force along the axial and radial directions (Figure 3C). The APC with 60% MXene exhibited the strongest mechanical strength when it was compressed along both axial and radial directions (3.005 kPa under 40% strain along the axial direction, and 2.127 kPa under 70% strain along the radial direction). In the two directions, the APC with 60% MXene also demonstrated the highest elastic modulus (1.59 and 0.83 kPa, respectively) with minimal plastic deformation (0.27%) (Figure 3D). These results indicate that the optimal ratio (60%) of MXene contents can produce the greatest number of hydrogen bonds to toughen APC. Notably, the aerogel of 100% MXene content exhibited complete brittleness, and its mechanical properties could not be evaluated due to structural fragility. The APC demonstrated high recoverability under uniaxial compression‐release cycles (Figure 3E). At low strain levels (ε < 60%), the unloading curves nearly overlap, indicating behavior within the elastic regime. At high strain levels (ε > 60%), the loading and unloading curves deviate, suggesting that the deformation cannot be immediately recovered upon unloading. However, after the first loading–unloading cycle, the APC retains a residual strain of less than 1% at 80% maximum strain. Table S2 (Supporting Information) summarizes the typical mechanical parameters of the APC. Additionally, Poisson's ratio (*ν*) was measured when the APC is subject to varying degrees of compression (Figure 3F). The results show that the APC exhibits a negative Poisson's ratio with a negative peak value of −0.496 and a convergence value of −0.19. A similar phenomenon was reported in previous studies.^[^ ^36^, ^48^ ^]^ This behavior arises from the multilayer stacked structure along the axis of the APC, where the unevenly distributed layers tend to compress and contact each other rather than separate or expand.

The anisotropic and multilayer stacked structures of the APC significantly enhance the alignment of electrical pathways, thereby enabling direction‐dependent piezoresistive. Electrical properties of the APC under in situ loads were characterized using a source measure unit (Figure 3A). We measured the relationship between the compression and the relative change in current, defined as (*(I‐I_0_)/I_0_*) along different directions of the APC. Sensitivity was calculated as the slope of the fitted linear curve of *(I‐I_0_)/I_0_*. Figure 3G exhibits that the APC enables directional‐dependent sensitivity. In the tests, radial compression was applied along the *y*‐axis, and sensitivity was measured at different orientations in the plane perpendicular to the load, the *z*‐axis was defined as 0°, and the *x*‐axis as 90°. As illustrated in Figure 3H,I, the APC exhibits the highest sensitivity at 0°, while the lowest sensitivity is observed at 90°. Additionally, the piezoresistive behavior of the APC can be categorized into three pressure regimes, with sensitivity decreasing to 284.3 kPa^−1^ in 700–2000 Pa at 90° and 96.9 kPa^−1^ in 800–2000 at 0°. This piezoresistive behavior is strongly related to the mechanical properties of the APC. Notably, the APC exhibits an ultrahigh sensitivity of 1055 kPa^−1^ at 0°, surpassing that of many previously reported sensors based on different sensing mechanisms (Table S3, Supporting information). The ultrahigh sensitivity is primarily attributed to the bioinspired structural alignment of high‐concentration TOCNs, highlighting its potential for directional sensing. Furthermore, we measured the sensitivity at 30°, 45°, and 60° in the three loading stages (Figure 3J). The results reveal that the sensitivity of the APC varies with direction. According to these values, the sensitivity can be expressed as a function of direction, suggesting its potential application in directional tactile sensing. Furthermore, the repeatability of the direction‐dependent piezoresistive behavior of the APC was also evaluated over 50 cycles, in which the sensitivity remains nearly constant across different directions (Figure S10, Supporting information). In addition, the limit of detection (LOD), the pressure‐resolution at an unloaded condition, is determined to be ≈1.5 Pa, an ultralow value that confirms the APC's excellent sensing capability (Figure S11, Supporting information).

Overall, the APC exhibits radial elasticity, ultrahigh sensitivity, and direction‐dependent piezoresistive behavior. These exceptional properties make it highly suitable for detecting tangential forces and microslip, as tangential loading induces deformation within multilayered stacked structures without compromising axial sensitivity.

### 2.4 Enhanced Tangential Sensitivity of the APC Sensor

**Figure 4:**
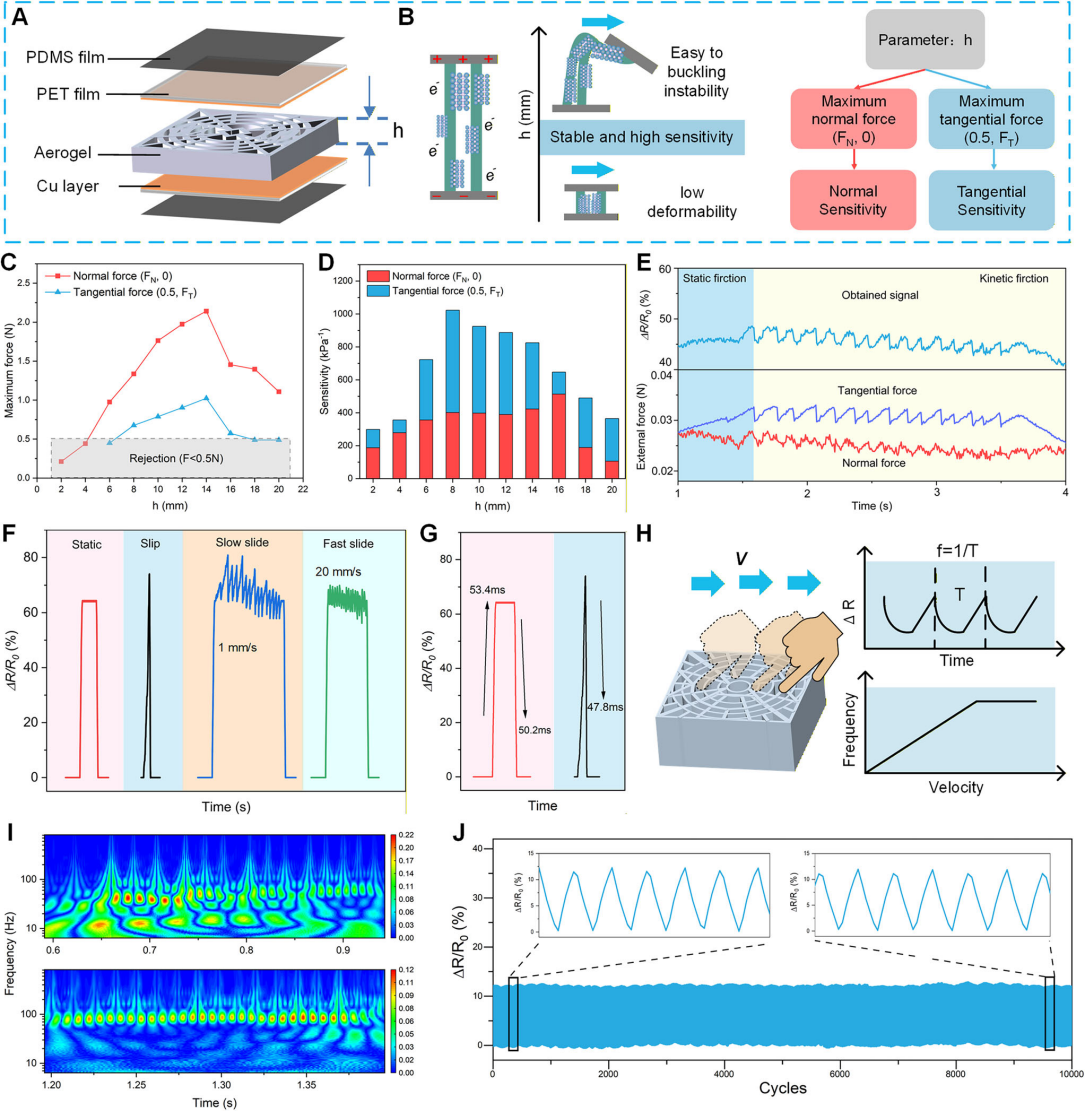
Enhanced tangential Sensitivity for stick‐slip tactile sensing of the APC sensor. A) Schematic diagram of the APC sensor structure. B) Illustration of the mechanism by which tangential sensitivity is modulated by the structural height (*h*) for determining an optimal height of the APC sensor (left). The sensitivity and load‐bearing capacity of the APC with different heights are examined under both normal and tangential forces (right). C) Maximum normal and tangential forces load‐bearing capacity, and D) the sensitivity for APC samples with different heights. The rejection region is highlighted on the graphs. E) The loading force (normal force and tangential force) and *ΔR/R*_0_ (%) signal of the tactile sensor measured during a complete period of sliding. F) The APC sensor responding to the static pressure, slip, slow (≈1 mm s^−1^), and fast sliding (20 mm s^−1^). G) Response and relaxation time of the APC sensor under static pressure and dynamic slip. H) Illustration of the relationship between slip velocity (*v*) and characteristic frequency (*f*). I) Time‐frequency mapping of the APC signals under slow (top) and fast (bottom) sliding. J) *ΔR/R*_0_ (%) of the APC sensor under 10 000 cycles of periodic loading (slip with a speed of 20 mm/s) for durability test.

A tradeoff between sensitivity and sensing range of the tactile sensor is important for ATSS.^[^ ^49^, ^50^ ^]^ Our APC sensor was designed to i) withstand the weight of common objects without structural failure, and ii) detect tangential forces and slip events with high sensitivity. **Figure** **4**A shows the structure of the APC sensor, which consists of a single layer with 60% MXene content, two flexible copper (Cu) electrodes deposited on polyethylene terephthalate (PET), and two flat polydimethylsiloxane (PDMS) films for encapsulation. The height (*h*), width (*w*), and depth (*d*) are crucial physical parameters of the APC sensor, among which *h* plays a dominant role in enabling lateral deformation along the X‐direction. We used 20 mm × 20 mm (*w* × *d*) freezing mold to ensure a uniform diffusion of the temperature gradient in the radial direction. The *h* of the APC could be conveniently adjusted by varying the volume of the MXene‐TOCNs dispersion. We used a single‐layer model representing the APC to illustrate how the height influences deformation under tangential loading (Figure 4B). Generally, a small *h* limits lateral deformation and relative displacement between lamellae. Conversely, a large *h* tends to induce buckling instability, potentially inducing structural failure and leading to a nonlinear sensing response. Therefore, an optimal *h* exists that allows tangential sensitivity while maintaining mechanical robustness. To confirm this hypothesis, ten APC sensors with varying heights (2–20 mm) were fabricated and encapsulated. The load‐bearing capacity and sensitivity were examined for both normal and tangential forces. The sensor is required to withstand a minimal load‐bearing capacity (0.5 N in both the normal and tangential directions) for daily use. Here, a constant normal force of 0.5 N was maintained (0.5, F_T_) to ensure an effective measurement of tangential forces. The normal force sensitivity is calculated based on the change of the resistance per unit normal force (S_(FN,0)_ = (R_(FN,0)_‐R_(0, 0)_)/F_N_) whereas the tangential force sensitivity is calculated as the change of the resistance per unit tangential force under 0.5 N normal force (S_(0.5, FT)_ = (R_(0.5, FT)_‐R_(0.5,0)_)/F_T_).^[^ ^49^ ^]^ Figure 4C shows the maximum normal and tangential forces that the APC sensor can withstand before the axial strain reaches 45%, beyond which an irreversible deformation could occur. When *h* is 14 mm, the APC exhibits the highest load‐bearing capacity, withstanding up to 2.141 N of normal force and 1.023 N of tangential force. However, beyond this height, the load‐bearing capacity decreases rapidly due to bulking, particularly for tangential loading. Samples falling within the shaded region were excluded due to failing to meet the minimal load‐bearing capacity (0.5 N). Tangential force and normal force sensitivities are closely correlated; and the tangential sensitivity is always higher than the normal sensitivity (Figure 4D). The resistance responses demonstrate that sensitivity increases with the height of the APC, confirming that the sensing sensitivity can be adjusted by the *h* of the stacked layers. We found that the APC with a height of 8 mm exhibits an optimal tradeoff: the tangential force sensitivity is close to 1022 kPa^−1^, while the normal force sensitivity is 401 kPa^−1^. Based on this analysis, the height of the APC sensor was fixed at 8 mm.

We evaluated the capacity of APC for distinguishing multidimensional tactile events. The relative resistance changes ratio (*ΔR/R*_0_) generated by normal and tangential force in both static and kinetic friction phases is collected and analyzed (Figure 4E). During the static phase, a noticeable increase in tangential force was observed, corresponding to a significant change in *ΔR/R_0_*. Upon exceeding the maximum static friction force, the slip occurred, leading to a sharp decrease in tangential force accompanied by the electrical signal jitter. These results indicate that the APC sensor can effectively capture the “stick‐slip” behavior. Figure S12 (Supporting Information) shows that the Pearson correlation coefficient between the resistance signal and tangential force (r = 0.84 ± 0.13) is higher than that with the normal force (r = 0.68 ± 0.19). Note that in the static friction, the coefficients were 0.86 ± 0.09 for tangential force, while 0.19 + 0.03 for normal force, indicating that the resistance signal accurately indicated the static friction while remaining largely independent of the normal force.

Table S3 (Supporting Information) lists a comprehensive comparison of mechanisms, structure, and sensitivity between our sensor and reported sensors.^[^ ^29^, ^51^, ^52^, ^53^, ^54^, ^55^, ^56^, ^57^, ^58^, ^59^ ^]^ Most existing sensors are sensitive to normal force or vertical deformation, allowing the high sensitivity to normal force, while their sensitivity to tangential forces is relatively low, which is common for many porous‐based sensors. This is due to the fact that porous structures fail to generate additional interfacial contact under tangential loading. Compared to these sensors, our APC sensor achieves high tangential sensitivity even under small normal forces since greater contact within the lamellar can be induced by tangential loading. This strategy effectively enhances the detection of tangential forces and “stick‐slip” state, even under subtle normal loading, which is exactly desired for multidimensional tactile sensing.

### 2.5 Stick‐Slip Sensing Capability of the APC Sensor

We examined the APC's response to four common tactile stimuli, including static contact, slip, static contact followed by slow slide, and static contact followed by fast slide, to assess its multidimensional sensing performance (Figure 4F). In static contact, the APC sensor exhibits pronounced changes in *ΔR/R*_0_ at both the onset and the offset of the applied stimulus, while remaining a stable signal during the sustained‐loading phase. In the slip event, the signal exhibits a transient single‐peaked response, with a peak magnitude exceeding that induced by static pressure, which is attributed to the APC's ultrahigh sensitivity to tangential forces. Under sliding conditions, the reactive signals showed clear differences depending on the sliding speed: fast sliding (20 mm s^−1^) generated responses with longer duration and higher frequency compared to slow sliding (1 mm s^−1^). More importantly, the signal baseline remains stable, indicating that the tangential response is not affected by the underlying static loading. The response‐relaxation time is a crucial parameter that determines the capability of a sensor to detect high‐frequency signals.^[^ ^50^ ^]^ The APC sensor exhibits a rapid response time of 53.4 ms and a relaxation time of 50.2 ms under static loading (Figure 4G). During slip, the relaxation time further decreases to 47.8 ms. The fast response and recovery behavior of the APC highlights the APC's capability to detect high‐frequency and dynamic tactile events.

The signals from “stick‐slip” tactile interaction are significantly affected by the sliding velocity (Figure 4H). When a finger or an object slides across the APC sensor surface at varying velocities (*v*), the periodic stick‐slip events are modulated by the *v*, resulting in a shortened signal period (*T*) at higher *v* while a prolonged signal period (*T*) at lower *v*. Consequently, the oscillating signal generated a dominant frequency defined by *f* = 1 / *T*. At the microscale, the *f* is further governed by the formula:

(1) f=v/λ

where *f* is the characteristic frequency, *v* is the sliding speed, and *λ* indicates the micro asperities (tiny peaks and valleys) of the surface.^[^ ^60^, ^61^ ^]^ In this study, *λ* is determined by the sensor–interface configuration; the *f* scales linearly with sliding speed, enabling identification of sliding velocity. Figure 4I presents time–frequency spectrograms of the APC response to slow and fast sliding. The frequency of *ΔR/R*_0_ changes significantly with sliding velocity: faster sliding induces higher‐frequency components (≈100–150 Hz) with shorter oscillation periods and stronger spectral concentration. This demonstrates the APC's potential for velocity‐sensitive slip detection characterized by spectral features. The signals in the frequency domain can be seen in Figure S13 (Supporting Information). Also, the relation between the signal frequency and the sliding velocity was further evaluated (Figure S14, Supporting Information). At low velocities, the data follow the theoretical model *f* = *v* / *λ*, but maintain stable when the velocity exceeds 20 mm s^−1^. This trend is consistent with previous physiological studies that a multiple‐fold increase in sliding velocity does not result in a proportional increase in the characteristic frequency.^[^ ^62^ ^]^ This suggests that high‐speed interaction on the skin surface consistently generates characteristic frequency signals that always fall into the identifiable range of PCs.

For practical application, the stability of the tactile sensor should be evaluated to ensure its reliability under repeated mechanical compressing and sliding. Here, the APC sensor was applied to cyclic sliding loads for 10 000 cycles (Figure 4J). No significant drift or degradation of the signals was found in the total duration. The excellent durability of the APC is attributed to the combination of radial elasticity and axial ultra‐sensitivity, allowing both mechanical reliability and high‐performance sensing. Compared to conventional tactile sensors composed of laminated individual layers, the APC sensor exhibits superior dynamic stability during prolonged interaction, making it a promising candidate for real‐world tactile sensing applications.

### 2.6 APC Array System Mimicking PC Clusters for Multidimensional Tactile Perception

**Figure 5:**
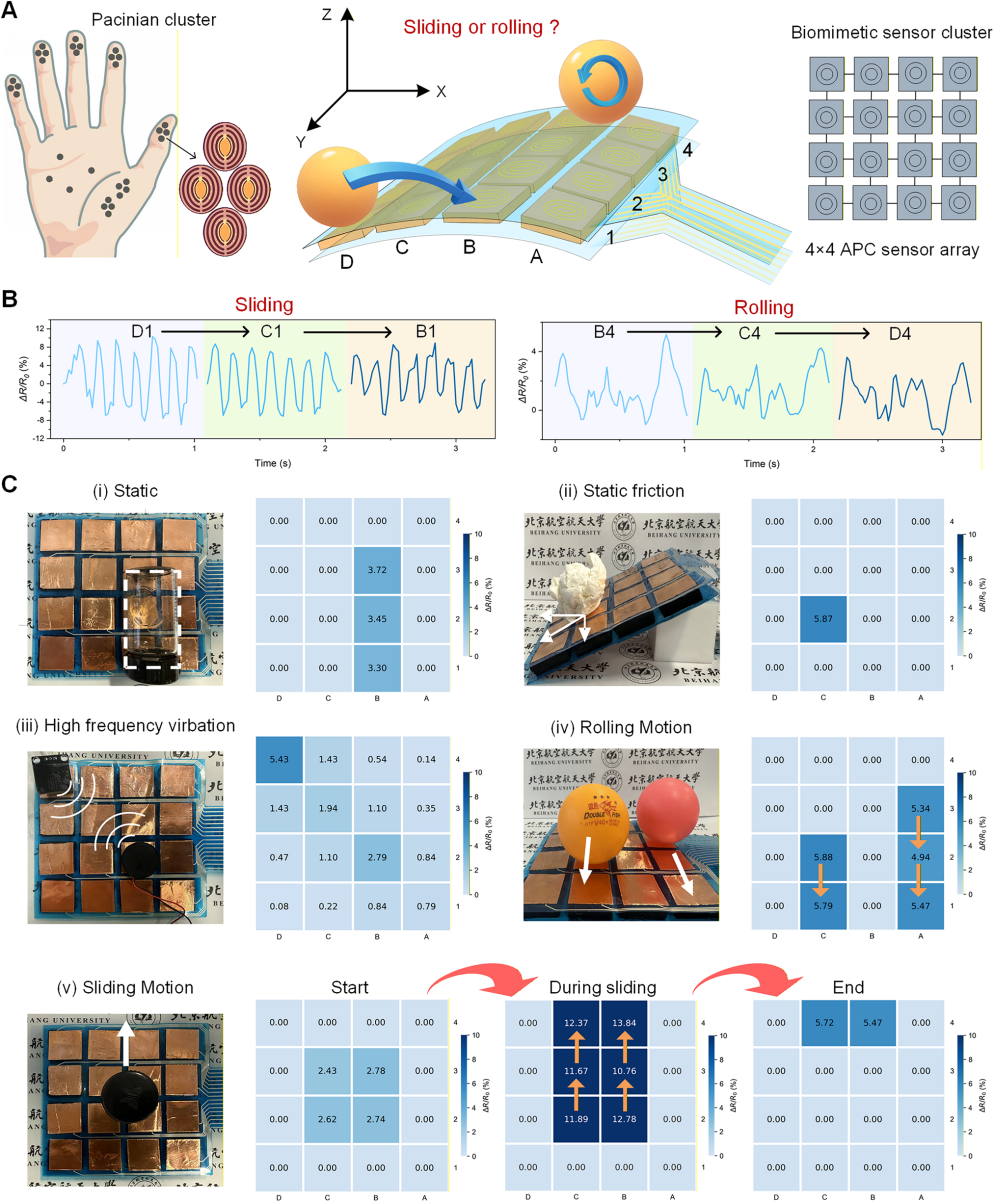
A tactile sensory system with an APC array for spatiotemporal sensing. A) Schematic illustration of a tactile system with 4 × 4 APC sensors mimicking PC clusters in human skin. B) The APC array distinguishes between sliding and rolling motions of a table tennis ball. C) Spatial responses indicating its capabilities in i) object shape recognition, ii) static friction detection, and iii) high‐frequency vibration sensing (D4: 40 Hz; B2: 20 Hz). Spatiotemporal responses of the APC sensory system for iv) real‐time tracking of rolling trajectories and v) monitoring directional sliding motions.

Human PCs appear in clustered arrangements, particularly in areas requiring high tactile acuity, such as fingertips and palms.^[^ ^17^, ^24^ ^]^ Such clustered configurations are highly desirable in tactile sensing,^[^ ^63^, ^64^ ^]^ as they facilitate the transmission of multidimensional information and enhance the stimuli recognition. In addition, unidirectional alignment of the PCs clustering is believed to support directional selectivity.^[^ ^24^, ^65^ ^]^ Mimicking the PC clustering, we fabricated a tactile system with 4 × 4 array sensors, with each sensor (20 mm × 20 mm × 8 mm, L × W × H) aligned uniformly along the axial direction. Copper foil electrodes were placed on both the top and bottom of each sensor. A PDMS double‐layer membrane with Ag wires was embedded to serve as a conductor for signal output. The APC sensory system was flexible and bendable, with ultralight weight ≈50 g (**Figure** **5**A). We examine the sensing capability of the system to interacting with lightweight objects, a table tennis ball (≈2.7 g). Two motion types of the ball, including sliding and rolling, were considered in this examination. The results indicate that two motion types were clearly differentiated by the APC array (Figure 5B). During sliding from D1 to B1, the signal exhibited shorter periodicity, larger fluctuations, and a pronounced stick‐slip feature. In contrast, during rolling from B4 to D4, the signal displayed longer periodicity and smoother variations. The maximum *ΔR/R*_0_ (10.31%) in sliding motion was significantly higher than that during rolling (5.14%). More importantly, as the ball moved across different APC units (e.g., started from D1, passed through C1, and finally arrived in B1), the corresponding signals responded with similar spatiotemporal patterns. By analyzing the spatiotemporal response, the sensor array could identify the cues of motion intent, current state, and trajectory of interacting objects.

As shown in Figure 5C, when the APC system senses an object larger than a single sensor i), multiple sensors are simultaneously activated. Through spatial mapping, the shape, weight, and the placing orientation of the object can be reconstructed. When the system is placed by a tilted angle ii), even a lightweight crumpled paper ball (≈3 g) generates sufficient static friction to activate tangential sensing, producing a pronounced signal response. This demonstrates that the APC system has the capability to detect subtle static friction, providing early warnings for potential slip events. Upon placement of a vibration motor iii), the system accurately localizes the vibration source. In addition to the primary unit detecting the stimulus, surrounding sensors capture the propagation of vibratory signals. Spatial signal mapping reveals the diffusion pattern of the vibrations, facilitating precise stimulus localization. When a table tennis rolling across the system iv), the APC sensors respond sequentially to encoding both temporal and spatial variations. This sequential activation pattern enables dynamic trajectory tracking with high resolution. During directional sliding motion v), the system captures a dynamic process when the relative motion onset. The initial contact position is first mapped, then gradually fades before reappearing at the final position. The time difference contains rich positional information, allowing precise spatial and temporal resolution of tactile stimuli. The increasing signal intensity with the object moving indicates that tangential components contribute to stronger responses. Abrupt rises and drops in signal magnitude correspond to the arrival and departure of the object from each sensor. Therefore, the spatiotemporal response of the APC sensory system can be used to detect object motion, direction, and interaction patterns, enabling robots to achieve rapid and accurate responses to dynamic tactile stimuli.

### 2.7 Multidimensional Tactile Sense Enhancing Human–Robot Interaction

**Figure 6:**
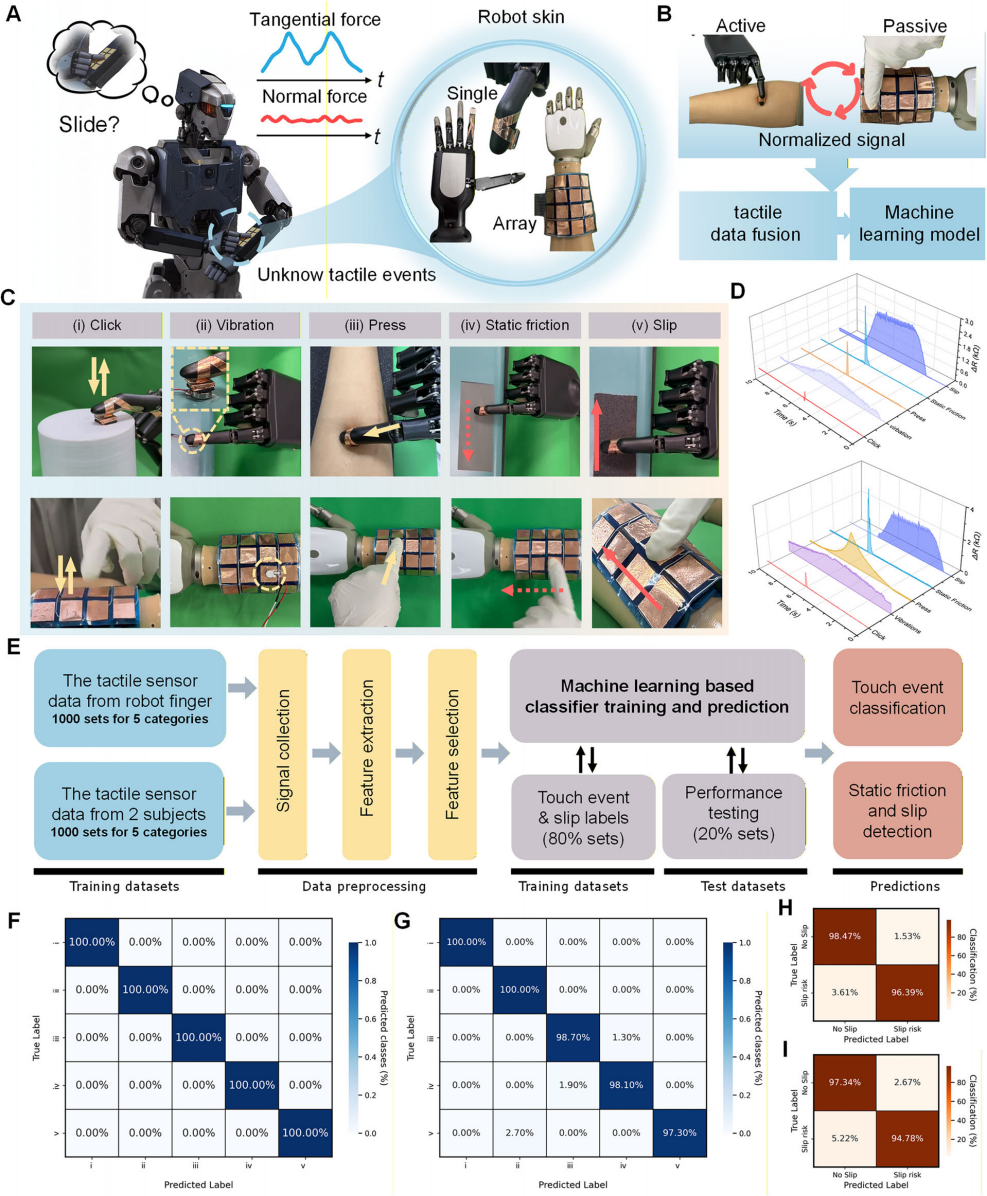
Multidimensional tactile sense enhances human‐robot interaction. A) Schematic diagram of a humanoid robot equipped with the APC system for multidimensional tactile sensing. B) A robotic finger with a single APC sensor acquiring active tactile signals and the robotic arm with an APC sensor array sensing passive tactile signals. Normalized signals are fused and labeled for the machine learning model. C) Five tactile events, including click, vibration, press, static friction, and slip, are detected in both active and passive tactile sensing. D) The resistance changes signal diagrams of different actions in human‐robot interaction. E) A flowchart illustrating the data processing and machine learning implementation for classifying tactile events and detecting static friction and dynamic slip. F) Confusion matrix for the classification of five tactile events during active interaction using the robotic finger, achieving 100% accuracy. G) Confusion matrix for the classification during passive interaction using the robotic arm, achieving an average accuracy of 98.82%. Confusion matrices for detecting static friction and dynamic slip are shown in (H) for active and I) for passive interactions, with average accuracies of 97.43% and 96.06%, respectively.

Sensitive multidimensional tactile sense enables robots to engage in physical and emotional interactions. Here, we examine the performance of the APC sensor and APC system in active and passive tactile interactions using a humanoid robot (**Figure** **6**A). Specifically, a single sensor was mounted on the robotic fingertip to actively acquire tactile information while an array of APC sensors was mounted on the robotic arm to passively sense tactile stimuli from humans (Figure 6B). Five representative tactile events (e.g., clicking, vibration, pressing, static friction, and slip) were included in this examination (Figure 6C). In general, the stimuli of click, press, and static friction elicit unimodal temporal responses, whereas vibration and slip produce oscillatory signal patterns (Figure 6D). We employed a multi‐channel data acquisition device to collect response signals, which were used to construct a tactile dataset. A machine learning model (Support Vector Machines, SVM) was used to classify tactile events characterized by complex features (e.g., force strength, friction direction, frequency) (Figure 6E). There are 10 categories in the dataset, each of which includes 200 samples. Prior to feature extraction, the data underwent a series of preprocessing steps, including signal filtering, normalization, and elimination of redundant components. Feature extraction was then performed using *Tsfresh*, a Python‐based toolkit that can automatically generate a comprehensive of descriptors from time‐series data. The features in the set refer to the time and frequency domain characteristics, statistical metrics, and wavelet transform‐based features Table S4 (Supporting Information). A five‐fold cross‐validation strategy was employed, in which each subset was used once as the test set while the remaining four served as training data. The classification of the test set was evaluated iteratively to ensure robustness. Figure 6F shows the confusion matrix for the classification results of the five tactile events in active sense. The results demonstrate that the APC sensor has excellent recognition performance for active tactile events, achieving an average of 100%. The high accuracy can be attributed to the high sensitivity of the APC sensor to subtle differences among events. The robotic finger performed motions with consistent force and velocity, facilitating reliable feature extraction. Figure 6G shows an average classification accuracy of 98.82% for passive tactile events, revealing good robustness and reliability. Compared to the active sense, a decrease in the classification accuracy of the passive sense is the result of inconsistent signals from human subjects who applied arbitrary forces to the APC array on the robotic arm. The incorrect identification primarily occurred in slow motion and slip detection, likely due to the inconsistent movement speed of the human hand. Notably, a robot equipped with APC sensors can selectively respond to tangential components before macroscopic motion begins, showing heightened sensitivity to static friction and incipient slip—a capability rarely reported in prior studies.

Further, datasets labeled as static friction and dynamic slip were grouped into one category, representing motion tendency and slip state. In contrast, click, press, and vibration were grouped into another category, labeled as no slip tendency. For active tactile sensing, the APC sensory system still achieves 97.43% average accuracy (Figure 6H), showing high performance for detecting unsafe manipulation. When the APC array senses passive tactile information from subjects, classification accuracy slightly decreases due to the diversity and complexity of the data (Figure 6I). However, the robot consistently achieves an average accuracy of 96.06%, resulting from the strong sensitivity of APC sensors to tangential components. We further fused data from both active and passive tactile signals and performed tactile event classification with 99.16% accuracy (Figure S15, Supporting information) and slip detection (Figure S16, Supporting information) with 96.74% accuracy. The outstanding sensing performance of the APC ensures consistently high accuracy, demonstrating its potential application as robotic skin. In particular, the APC sensor is characterized by ultralight weight to eliminate the inertial effect from additional accessories. Overall, the robot with the APC sensor and APC system can integrate features from both active and passive tactile sensing, enabling perception comparable to that of humans.

## 3 Discussion

In summary, our work reported a PC‐inspired artificial tactile sensory system with tangential sensitivity, frequency discrimination, and slip detection capabilities in multidimensional tactile perception and human–robot interaction. The system based on the APC is inspired by the multilayer stacked, anisotropic structure and the tangential sensitivity mechanism of human PCs. This biomimetic strategy generates an ideal integration of ultralight (7.781 mg cm^−3^) and ultrasensitive (1022 kPa^−1^) properties in the APC sensor. The tangential sensitivity is superior to previously‐reported tactile sensors to the best of our knowledge. The APC sensor has the capability to recognize multidimensional tactile stimuli comparable to human tactile perception. Also, the APC sensory system allows robots to precisely sense object shape, vibration, and friction and to accurately identify sliding and rolling motions. The APC sensory system can be mounted on humanoid robots via its adjustable size and deformability, without significant weight gain. A robot equipped with the APC system achieved accurate identification of tangentially related tactile events—including static friction and dynamic slip during both active and passive interactions, with accuracies of 100% and 98.82%, respectively. This capability advances robotic tactile perception and compensates for the lack of tangential sensing functions in existing tactile sensory systems, showing great potential for intelligent recognition and human–robot interaction. In future work, array density can be increased by downsizing individual sensing units, while upgrading on‐array multiplexing, data‐transmission bandwidth, and on‐board processing to support higher spatiotemporal resolution.

Our work contributes to understanding the mechanism of PCs and exemplifies how biomimetic research can provide bidirectional inspiration between biology and engineering. Firstly, the structure characterized by multilayered stacking and anisotropy enables radial elastic deformation and high axial sensitivity of the APC. This mimics the function of PCs, which perceive tactile stimuli through the deformation of radially distributed lamellar cells and transmit electrical signals via axially aligned synapses. Secondly, the multilayered stacked structure of the APC exhibits interlayer contact under tangential loading, reflecting the tangential sensitivity of PCs, which arises from increased stress on the nerve fiber membrane. Also, the APC sensor can detect frequency components from stick–slip state, achieving PC‐like function of slip detection based on high‐frequency cues. Our study integrated a tactile sensing paradigm to pioneeringly reveal the intrinsic linkage between tangential sensitivity, frequency discrimination, and slip detection. We reasonably hypothesize that a similar sensory coupling mechanism may have been evolutionarily retained in PCs. Thus, this biomimetic work exemplifies a bidirectional enhancement between technological innovation and biological insights.

## 4 Experimental Section

### Materials

Cellulose cotton linter (Ash‐less filter pulp, Beijing, China) was used as a native cellulose sample. 2,2,6,6‐tetramethylpiperidin‐1‐oxyl (TEMPO), sodium bromide, and sodium hypochlorite were purchased from Aladdin Co., Ltd., China. Ti_3_AlC_2_ powder was provided by Jilin 11 Technology Co., Ltd. (China). Lithium fluoride (LiF) was purchased from Aladdin (China). Sulfuric acid (H_2_SO_4_) and hydrochloric acid (HCl) were bought from Beijing Chemical Reagents Co., Ltd. (China). All the chemical reagents and solvents were of laboratory grade, and used without further purification. Distilled water filtered using a purification system was used throughout.

### Preparation of 2,2,6,6‐Tetramethylpiperidin‐1‐oxyl (TEMPO) Mediated Oxidized Cellulose Nanofiber (TOCN) Sol

Briefly, 0.33 g of NaBr and 0.033 g of TEMPO, accurately weighed, were added to a 400 mL volume of deionized (DI) water at 10 °C under vigorous stirring until completely dissolved. Then, 2 g of cellulose cotton linter and NaClO solution (15 mmol g^−1^ cellulose mass) were added to the obtained solution, where NaClO was employed to trigger the onset of the oxidation reaction. The pH was maintained at 10.5 by constant drops of 1 m NaOH solution in the reaction system for the next 6 h. Next, the oxidized nanocellulose was filtered and washed with DI water several times to neutralize the pH. The acquired oxidized nanocellulose was dispersed to prepare a 3 wt.% suspension, and the homogenization was performed six times at a pressure of 70 MPa. The final concentration of the TOCN colloidal suspension was adjusted to 1 wt.%. The as‐prepared dispersion is completely transparent.

### Preparation of Ti_3_C_2_T_X_ Nanosheet

Titanium carbide (Ti_3_C_2_T_x_) MXene nanosheets were synthesized by selectively etching Ti_3_AlC_2_ MAX phase powder (400 mesh) with a LiF/HCl solution, using the minimally intensive layer delamination (MILD) method.^[^ ^56^ ^]^ Detailed fabrication procedures are provided in the Supporting Information.

### Preparation of TOCNs/MXene Aerogel (APC)

The aerogel was prepared using an improved multidirectional freeze‐drying method. In a typical experiment, a certain amount of the TOCNs dispersion (1 wt.%) and MXene dispersion (20 mg mL^−1^) were mixed with a predetermined mass ratio. Then, the mixture of TOCNs and MXene was stirred and sonicated for 1 h. The uniform suspension was poured into a custom mold. The top of this mold is made of PTFE material to form a hollow square ring with a size of 25 × 25 mm, and the wall thickness is 2 mm. The bottom is made of a 100 mm long copper block as a thermal conduction element, and the two parts can be tightly inserted together. When the preparation process begins, the end of the copper block was inserted into liquid nitrogen (−196 °C) in a customized hemispherical Dewar bottle, and the other end used room temperature to form a temperature gradient in the vertical direction. Simultaneously, ice packs were placed around the mold to provide a temperature gradient from the outside to the inside, the cooler four sides as the cold source, and the inside of the samples as the heat source. After ≈10 min, the liquid sample has frozen into a hard solid. Finally, the samples were freeze‐dried at −10 °C for one day, then at −5 °C for one day, and finally at 0 °C for 72 h to preserve the microstructure from being damaged by physical forces. During the experiments, we also prepared aerogels with different mass ratios of TOCN and MXene, setting up control experiments. These aerogels were prepared using the same method, but with different TOCN/MXene ratios (Table S1, Supporting Information). We also prepared aerogel using conventional freezing methods to gain a deep understanding of the PC inspired structure. The TOCN/MXene dispersion was placed into a −4 °C freezer for 6 h until the liquid was completely frozen. Subsequently, the samples were freeze‐dried for 5 days in a freeze dryer to obtain random aerogel.

### Preparation of APC Sensor

A 100‐nm‐thick layer of Cu was deposited onto a ≈50‐µm‐thick PET film (HD, 3 m) using ion sputtering (JFC1600, JEOL), which acted as the electrodes for the APC. Then the PET‐Cu film was further cut into 20 mm × 20 mm squares for subsequent use. The tactile sensor comprised five layers arranged from top to bottom, featuring a symmetrical “sandwich” structure: two polydimethylsiloxane (PDMS) membranes (≈80 µm) as the top and bottom encapsulation materials, two PET‐Au films serving as the top and bottom electrodes, and the APC positioned in the middle as the active sensing layer. The PDMS membranes were pretreated with plasma and bonded to the exposed sides of the PET‐Cu layers. Finally, the PDMS membranes were affixed using silicone adhesive (Sil‐Poxy, Smooth‐On, Inc.).

### Characterization and Measurements

The morphology of the TOCN was characterized using the high‐resolution transmission electron microscope (HRTEM) (JEM‐1400, JEOL, Japan) at 50 kV. The morphologies of MXene and APC were examined by field‐emission scanning electron microscopy (SEM, JSM‐7100F, JEOL, Japan), equipped with energy‐dispersive X‐ray spectroscopy (EDS) for elemental analysis. Chemical composition of the samples was investigated by Fourier transform infrared spectroscopy (FT‐IR, Bruker Tensor II, Germany). Images of the compression process were captured using a metallographic microscope (BX51, Olympus, Japan). Compressive tests of the APC were conducted on a universal testing machine (AGS‐X, SHIMADZU, Japan) equipped with a 100 N load cell at a strain rate of 3 mm min^−1^. Five replicate tests were performed for each sample series. Young's modulus was calculated from the initial 30% strain region. Poisson's ratio was determined as the ratio of lateral to longitudinal strain during compression. Electrochemical properties of the TOCN/MXene aerogel were evaluated using an electrochemical workstation (PGSTAT101, Metrohm Autolab, Netherlands). Signals from the tactile sensor were recorded using either a source measurement unit (SMU, 2602, Keithley, USA) or a digital data acquisition system. A vibration generator (3.7 V) was used to apply vibrations of constant frequency to the sensor, and corresponding response signals were collected via the digital acquisition system.

### Construction of the APC Sensor Array

The aerogel was fabricated to dimensions of 20 × 20 × 8 mm for the tactile sensor by controlling the volume of TOCN/MXene solution poured into the mold and trimming the TOCN/MXene blocks while frozen. The encapsulated sensor array comprised 16 individual sensors. Copper wires were connected to the electrodes of each sensor and routed to a printed circuit board (PCB) for real‐time resistance data acquisition, forming a 4 × 4 sensor array for data collection and transmission. The PCB acquires resistance signals using a time‐division multiple access (TDMA) method, and the collected data are transmitted to a computer for further processing.

### The Statement for Human Subjects

All authors have obtained a disclaimer of signed informed consent from the person who participated in our tactile sensor testing experiments with human subjects.

### Statistical Analysis

The meaning of all error bars and how they were calculated is described within the captions of the figures in which they occur.

## Conflict of Interest

The authors declare no conflict of interest.

## Supporting information



Supporting Information

## Data Availability

The data that support the findings of this study are available from the corresponding author upon reasonable request.
