# Supplementary material for: A Tangentially Sensitive Tactile Sensor Reveals the Stick‐Slip Mechanism and Enhances Robotic Tactile Sensing
Source: Adv Sci (Weinh). 2025 Nov 20;13(13):e17884. doi: 10.1002/advs.202517884 (PMC12955882; doi:10.1002/advs.202517884)
Supplement: Supplementary file 1 — Supporting Information [file ADVS-13-e17884-s001.docx]

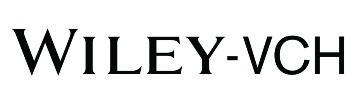


Supporting Information for

**A Tangentially Sensitive Aerogel Tactile Sensor Reveals the Stick–Slip Mechanism and Enhances Robotic Tactile Sensing**

Jinghui Wang *et al.*

*E-mail: x.y.liu@buaa.edu.cn (Prof. Xiaoyu Liu); lizhenwang@buaa.edu.cn (Prof. Lizhen Wang); yubofan@buaa.edu.cn (Prof. Yubo Fan).

**This file includes:**

Supporting Text

Supporting Figure S1 to S16

Supporting Tables S1 to S4

References (1 to 10)

Supporting Method

*Preparation of Ti_3_C_2_T_X_ Nanosheet*: First, 1.6 g of LiF was dissolved in 9 M HCl in a Teflon container under magnetic stirring for 10 min to ensure the mixture turned clear and transparent. Then, 1 g of Ti_3_AlC_2_ powder was added into the LiF/HCl etching solution slowly and the mixture was reacted at 35 °C under mechanical stirring for 36 h. The resulting Ti_3_C_2_T_x_ suspension was washed several times with DI water and centrifuged at 5000 rpm for 5 min until pH of the supernatant exceeded 5. The multilayered Ti_3_C_2_T_x_ was exfoliated by ultrasonication in a water bath and protected by nitrogen, followed by centrifugation for 1 h at 3500 rpm to obtain a dark green homogeneous supernatant with delaminated Ti_3_C_2_T_x_ nanosheets. To determine the concentration of the delaminated Ti_3_C_2_T_x_ nanosheet solution, a known volume of solution was filtered through a polypropylene filter (Celgard 3501 coated PP) and the solid content after vacuum drying was measured.

*Mobile platform for normal and tangential force sensing*: A custom apparatus was developed to provide shear force and slip events, which consists of a dual-axis electric drive stage (FMC 4030) and a commercial three-dimensional force sensor (iDAS R&D). The electric drive stage can move left and right along the X-axis while moving up and down along the Z-axis. A three-dimensional force sensor is fixed to the moving platform, allowing for simultaneous detection of forces from three different directions. The pressure applied on the tactile sensor was controlled by adjusting the height of the Z-axis. The sensor slid across the surface of each object in the X-axis to interact with its surface and corresponding resistance signals were recorded. Prior to each test, the sample was firmly attached to a stationary base using double-sided adhesive tape to prevent any undesired movement.

Supporting Figure


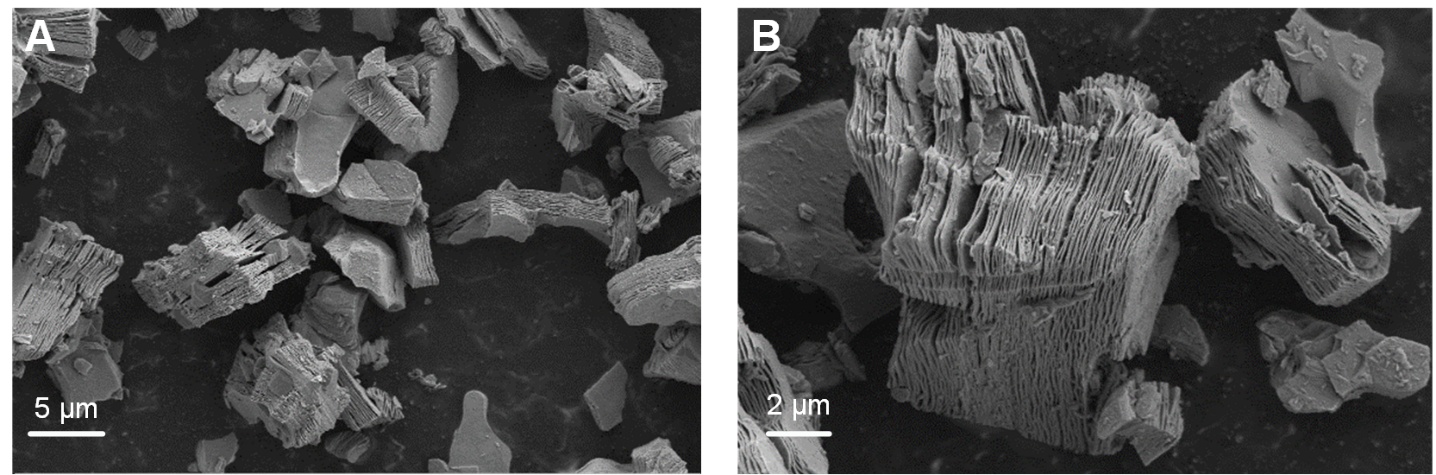


Figure S1. Structural SEM images of MXene. (A) Overview showing the typical layered and accordion-like morphology of delaminated MXene flakes. (B) Higher magnification image highlighting the ultrathin lamellar structure and surface roughness of individual flakes


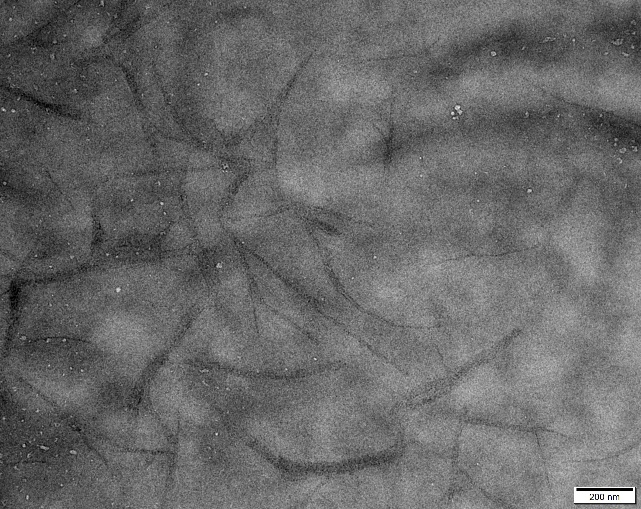


Figure S2. TEM image of TEMPO-oxidized cellulose nanofibers (TOCNs). The image shows well-dispersed, long, and entangled one-dimensional nanofibrils with diameters below 10 nm.

**
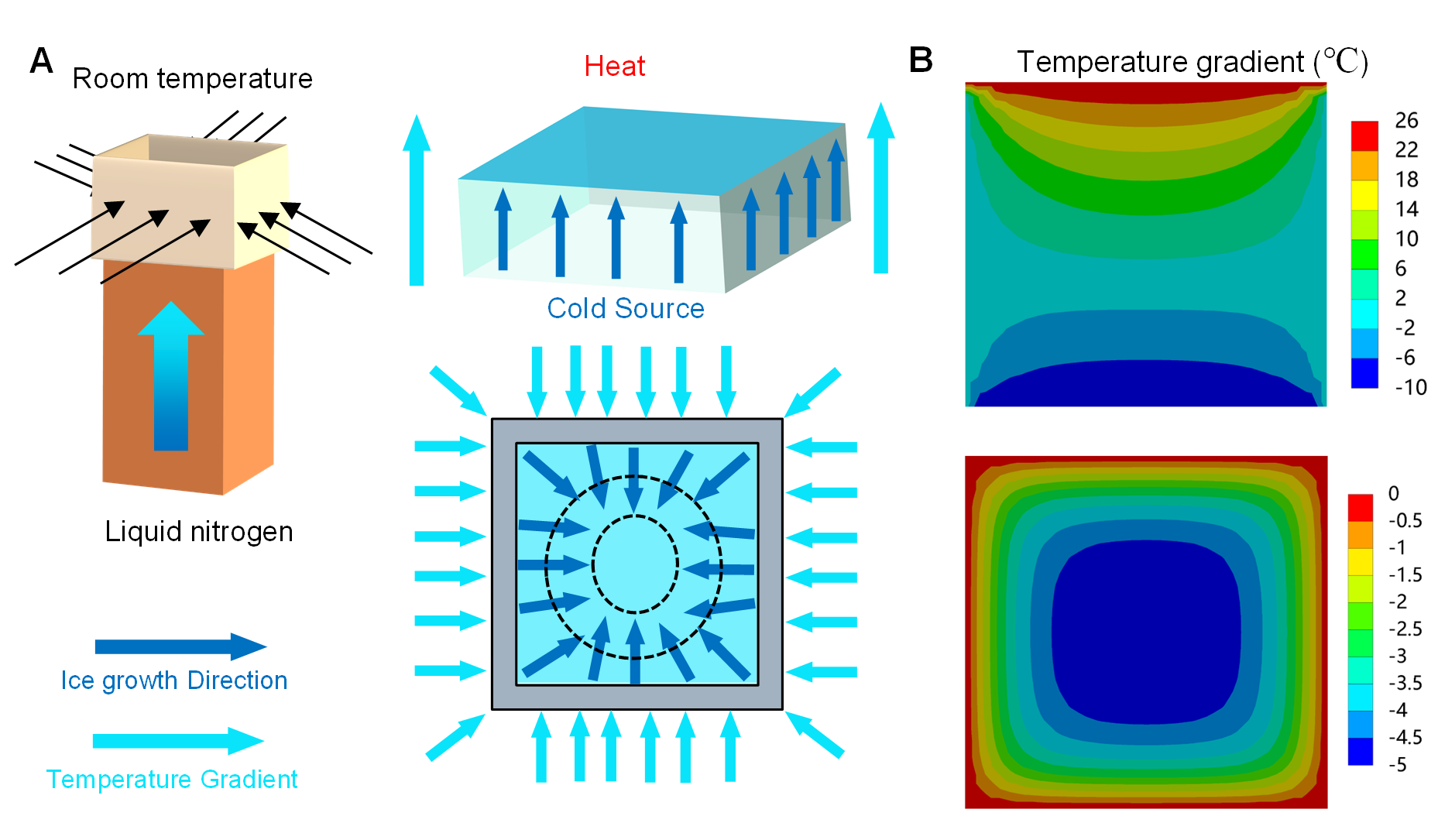
**

Figure S3. Schematic illustration of the fabrication of APC with multilayer stacked and anisotropy by directional freeze-drying process. (A) The axial (bottom-to-top) and radial (outside-to-inside) temperature gradients guide ice growth to form the anisotropic lamellar structure. (B) The FEA results of the temperature gradient.


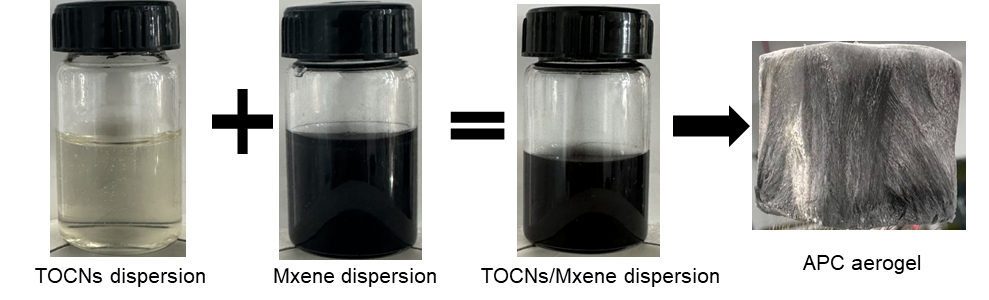


Figure S4. Photographs of materials and gelation process of the fabrication of the APC. From left to right: aqueous suspension of TEMPO-oxidized cellulose nanofibers (TOCNs); dispersion of Ti₃C₂T_x_ MXene nanosheets; homogeneous TOCN–MXene mixed dispersion; and the resulting ultralight, freestanding APC aerogel after multi-directional freeze-drying.

**
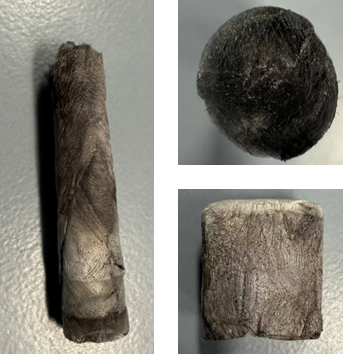
**

Figure S5. Photographs of APC aerogels with different shapes fabricated by varying mold designs. The results demonstrate the structural integrity and shape customizability of the APC.


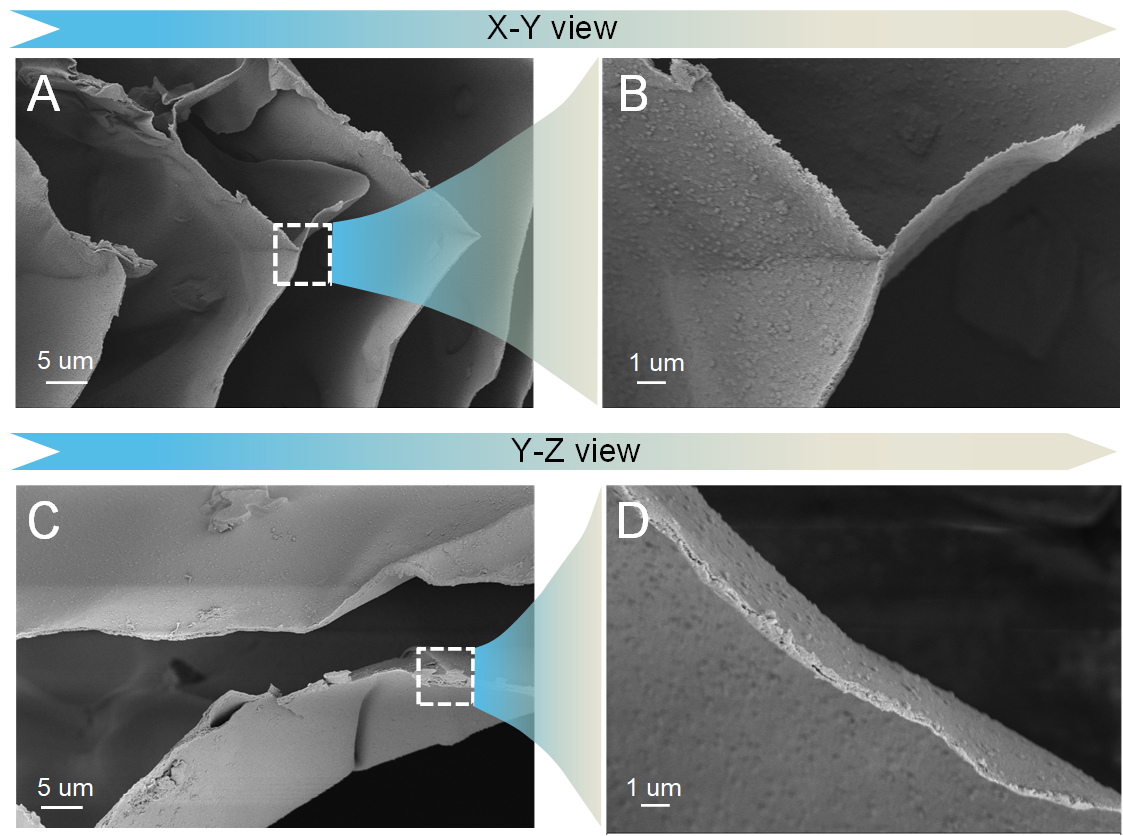


Figure S6. SEM images of the APC structure at different magnifications. (A) and (B) X–Y view showing the smooth lamellae with edges. (C) and (D) Y-Z view revealing the extended, continuous layered structure with clear interlamellar interfaces.


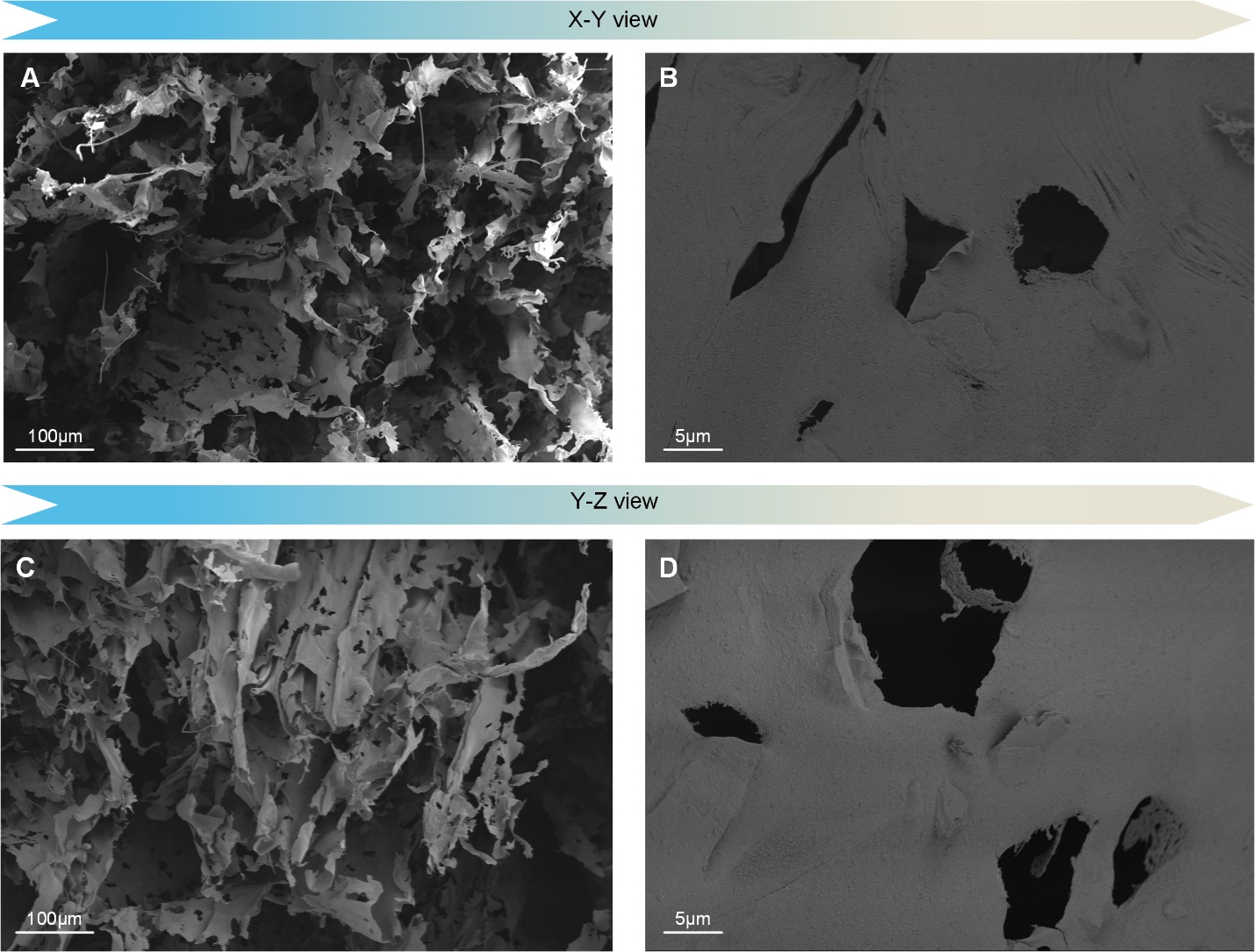


Figure S7. **SEM images of the random structure at different magnifications.** (A) and (B) X–Y view showing a disordered, isotropic pore network with irregular lamellae and open pores. (C) and (D) Y–Z view revealing similarly disordered morphology without alignment.


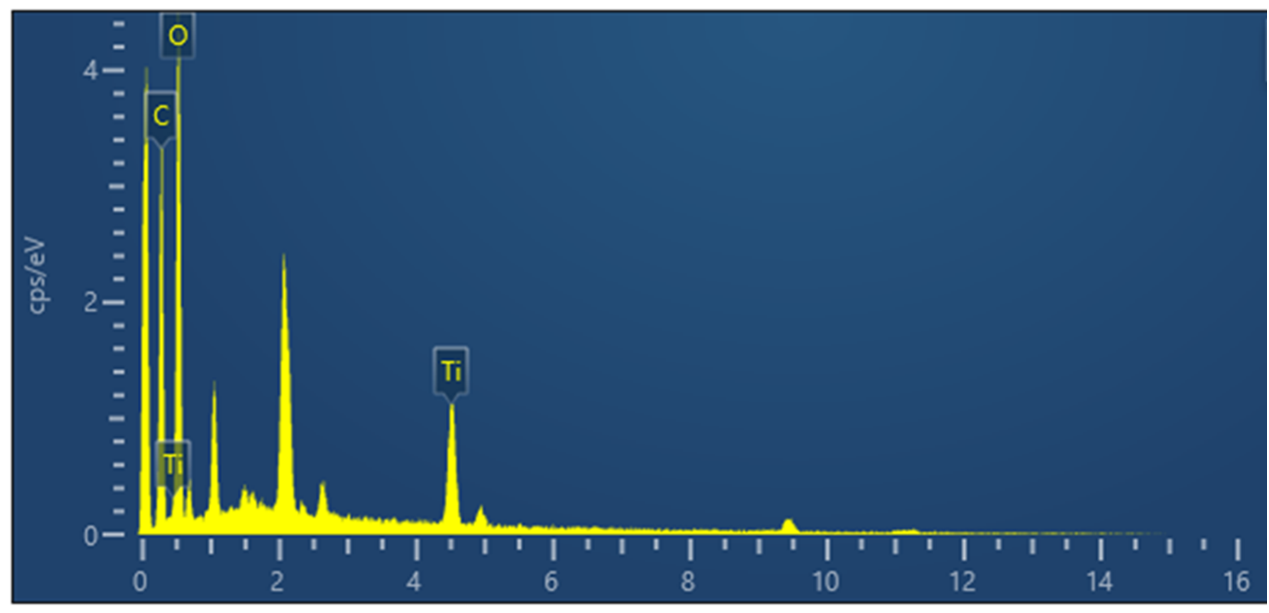


Figure S8. EDS spectrum obtained from the selected area of the APC. The presence of Ti, C, and O elements corresponding to the MXene (Ti₃C₂Tₓ) and TOCN components.


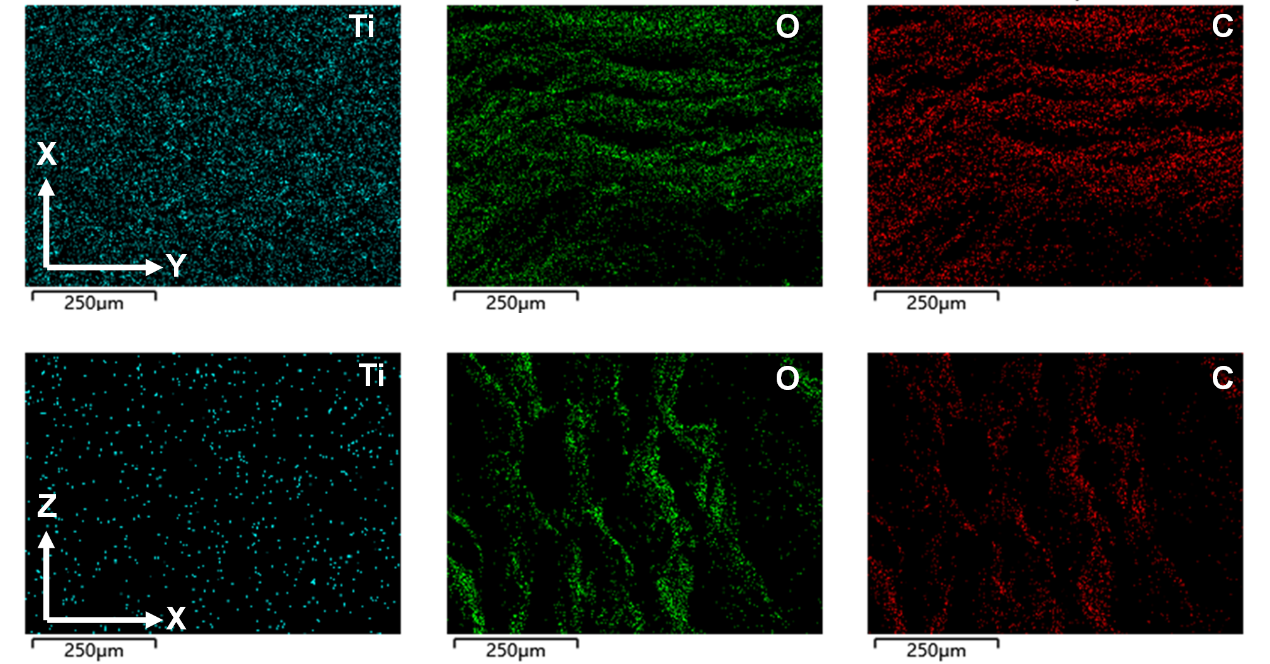


Figure S9. Elemental mapping images of Ti, O, and C in the APC aerogel along two orthogonal planes. Elemental distributions of Ti, O, and C in the X–Y plane, revealing uniform in-plane dispersion of MXene and TOCN components and corresponding elemental mappings in the X–Z plane.

**
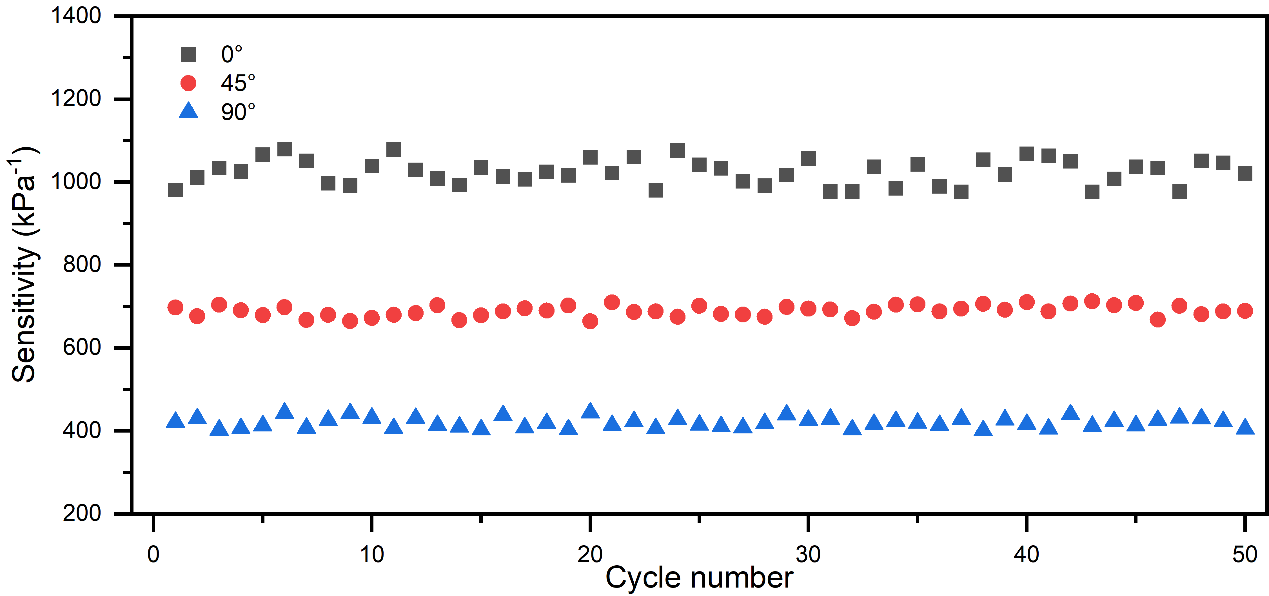
**

Figure S10. Sensitivity of the APC sensor under repeated loading cycles (0–40 Pa) at different directions. Sensitivity was measured over 50 cycles at 0°, 45°, and 90° orientations. The results show stable and direction-dependent responses.


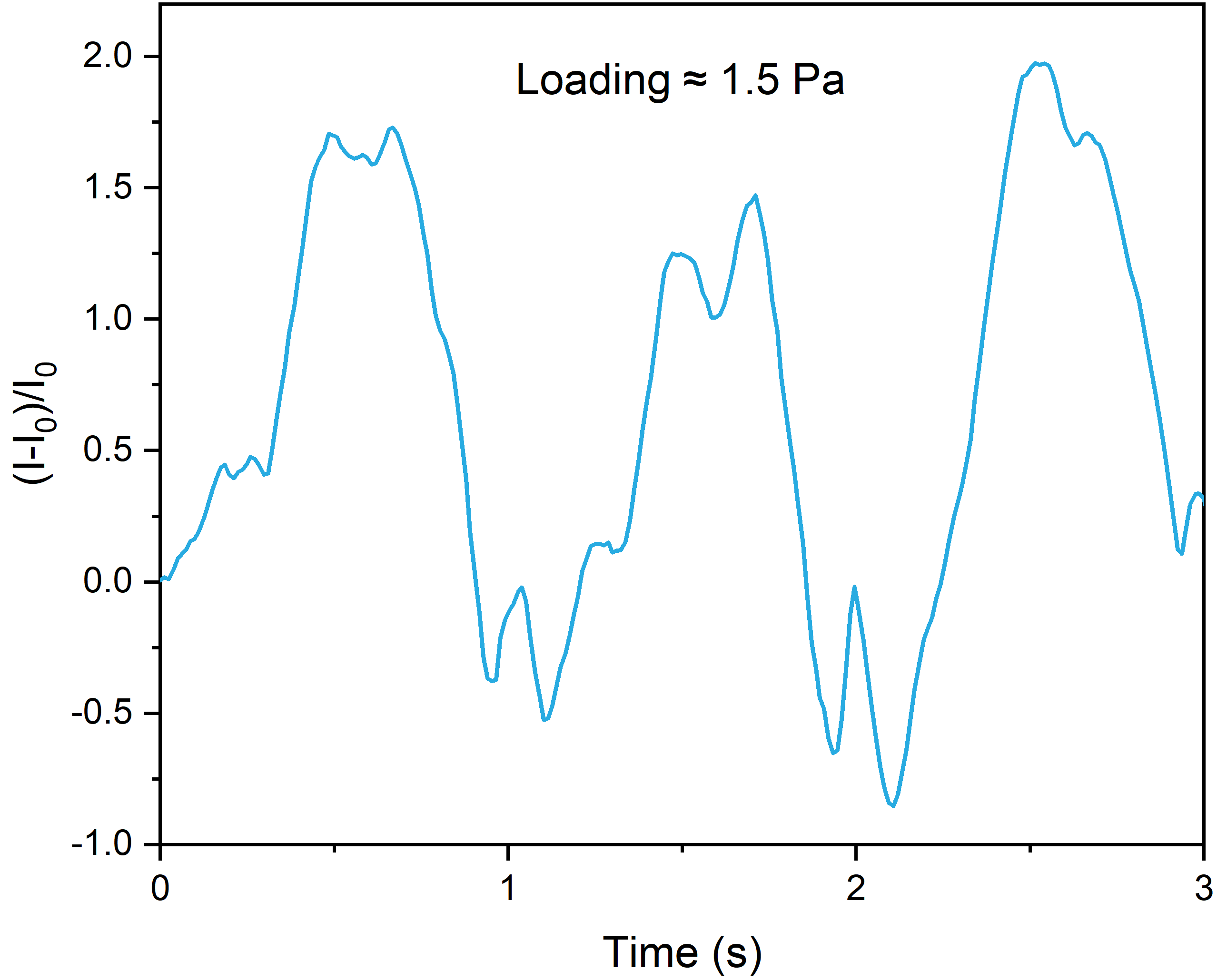


Figure S11.  Limit of detection of the APC. Time-resolved normalized current response of the APC under a low amplitude pressure stimulus of 1.5 Pa.

**
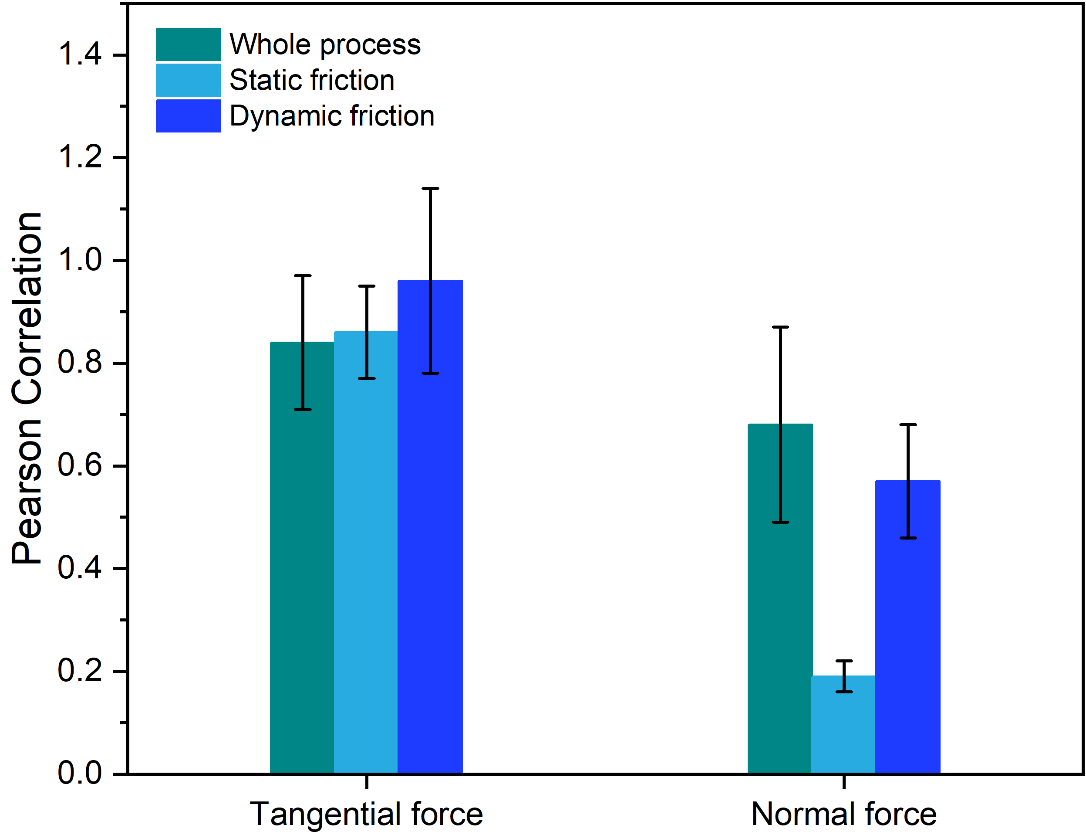
**

Figure S12. Pearson correlation coefficients between resistance signals and applied forces during different frictional stages. The correlation was calculated for both tangential and normal forces over the whole process, static friction phase, and dynamic friction phase. The results show consistently higher correlation with tangential forces, particularly during dynamic friction, indicating that the APC is more sensitive to shear-related tactile stimuli than to normal pressure.


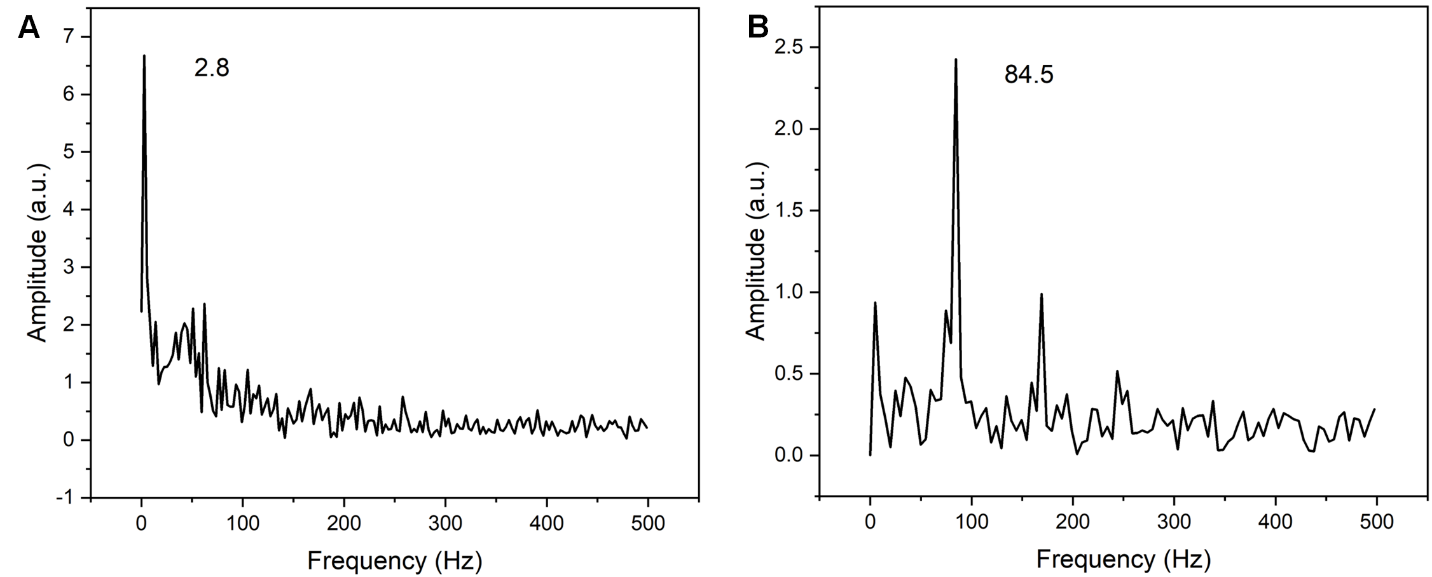
 Figure S**13. Characteristic peaks of APC signals under slow and fast sliding motions in frequency domain.** (A) the APC signal under slow sliding (1 mm/s), showing a dominant frequency at 2.8 Hz. (B) the APC signal under fast sliding (20 mm/s), showing a dominant frequency at 84.5 Hz.


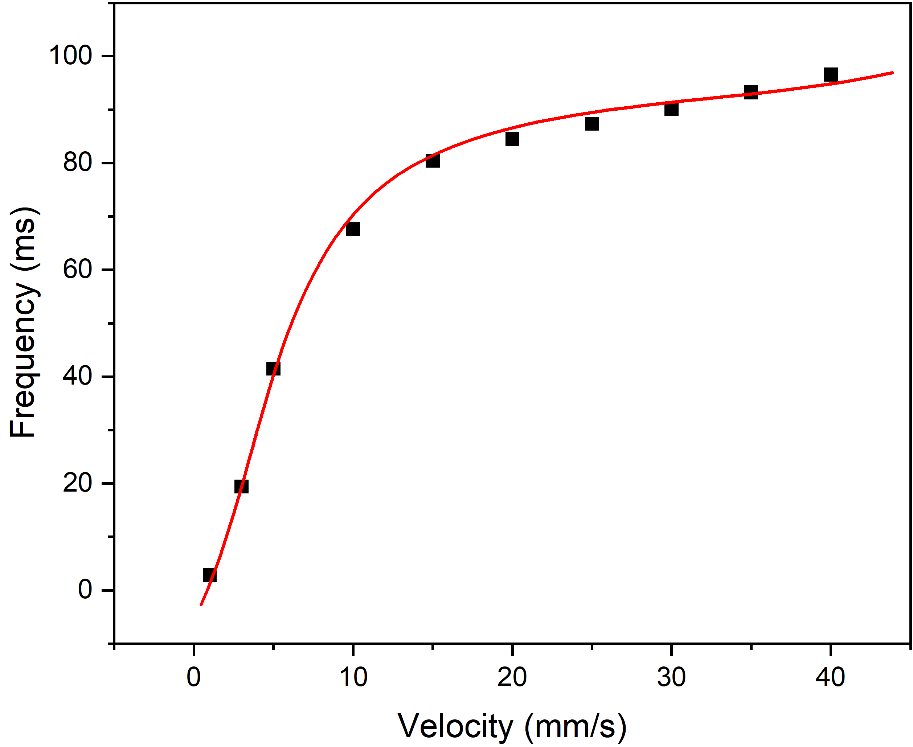


Figure S14. Relationship between characteristic frequency and sliding velocity of the APC sensor. The results demonstrate that the characteristic frequency increases with velocity and gradually approaches a saturation value, reflecting the APC’s ability to encode slip velocity through frequency-modulated electrical responses.


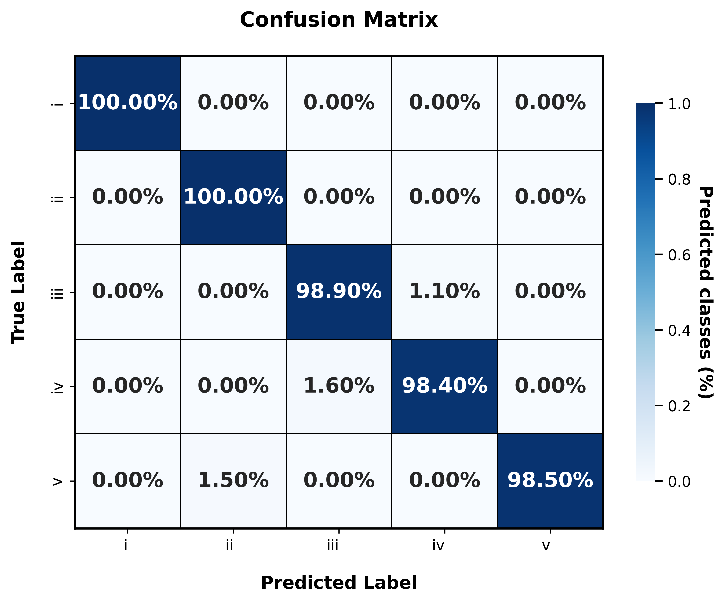


Figure S15. Confusion matrix for tactile event classification using fused active and passive tactile data. The classifier achieved an average accuracy of 99.16%.


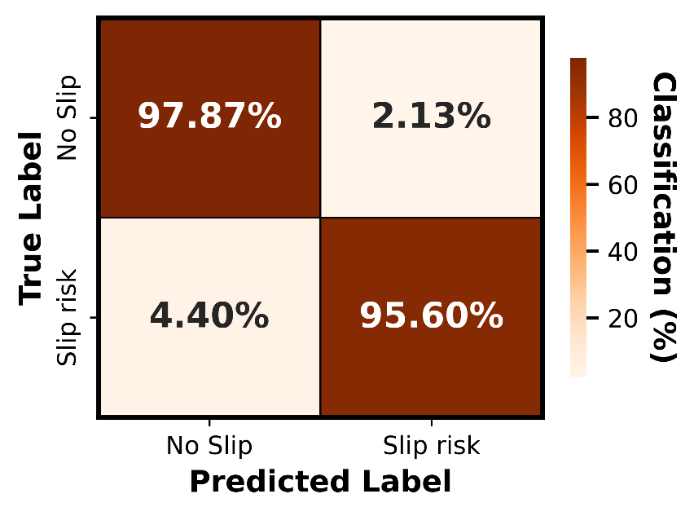


Figure S16. Confusion matrix for slip risk detection using fused active and passive tactile data. The classifier accurately achieving an average accuracy of 96.74%.

**Supporting Table**

Table S1. Raw Materials for TOCN/MXene Aerogel Preparation

| V _TOCN_ (mL) | V _MXene_(mL) | V _Water_(mL) | M_TOCN_/M_MXene_ | M _TOCN_ (mg) | M_MXene_ (mg) |
| --- | --- | --- | --- | --- | --- |
| 0 | 5 | 5 | 0:10 | 0 | 100 |
| 2 | 4 | 4 | 2:8 | 20 | 80 |
| 4 | 3 | 3 | 4:6 | 40 | 60 |
| 6 | 2 | 2 | 6:4 | 60 | 40 |
| 8 | 1 | 1 | 8:2 | 80 | 20 |

Table S2. Summary of the mechanical properties of the APC

| M_TOCN_/M_MXene_ | Loading Direction* | Max compress stress (kPa) | Compressive modulus (kPa) | Toughness (kPa) | Plastics deformation (%) | Dissipated energy (kPa) |
| --- | --- | --- | --- | --- | --- | --- |
| 0:10 | Axial | - | - | - | - | - |
|  | Radial | - | - | - | - | - |
| 2:8 | Axial | 1.175 | 1.12 | 13.692 | - | - |
|  | Radial | 1.070 | 0.42 | 21.624 | 24.21 | - |
| 4:6 | Axial | 3.005 | 1.59 | 25.545 | - | - |
|  | Radial | 2.127 | 0.83 | 37.370 | 0.27 | 8.654 |
| 6:4 | Axial | 2.499 | 1.47 | 23.045 | - | - |
|  | Radial | 1.419 | 0.72 | 28.350 | 1.45 | - |
| 8:2 | Axial | 1.687 | 1.37 | 17.890 | - | - |
|  | Radial | 1.209 | 0.65 | 25.189 | 2.80 | - |

Table S3. The comparison of performance of existing sensors.

| Sensor type | Materials | Mechanism | Sensitivity (kPa^-1^) | Stability (cycles) | Density (mg/cm^3^) | Response time (ms) | Ref. |
| --- | --- | --- | --- | --- | --- | --- | --- |
| Aerogel | Graphene | Piezoresistive | 92.22 | 6000 | - | 11 | 1 |
| Film | Graphene | Piezoresistive | 10.39 | 1100 | - | 11.6 | 2 |
| Film | Carbon nanotubes | Piezoresistive | 10.55 | 500 | - | 216 | 3 |
| Film | Carbon nanotubes | Piezoresistive | 3.74 | 5000 | - | 15 | 4 |
| Microparticles | ZnO | Piezoresistive | 94 | 2000 | - | 16 | 5 |
| Sponge | MXene | Piezoresistive | 147 | 10000 | - | 138 | 6 |
| Aerogel | MXene | Piezoresistive | 24.63 | 5000 |  | 14 | 7 |
| Film | rGO | Piezoresistive | 0.82 | - | - | 24 | 8 |
| Transistors | 2D semiconduct | Transistor-based capacitive | 770 | - | - | 0.1 | 9 |
| Capacitance | H_3_PO_4_ | Iontronic | 229.9 | 5000 | - | 9 | 10 |
| **This work** | MXene | Piezoresistive | 1022 | 10000 | 7.781 | 53.4 | - |

Table S4. Top ten important extracted signal features

|  | **Feature Name** | **Description** |
| --- | --- | --- |
| 1 | Sum of reoccurring data points | Sum of duplicate values |
| 2 | Longest strike above means | Longest above-mean streak |
| 3 | FFT coefficient attr "real" coeff | Real part of FFT coefficients |
| 4 | FFT coefficient attr "abs" coeff | Magnitude of FFT coefficients |
| 5 | Sum values | Sum over the time series values |
| 6 | Skewness | Asymmetry of the distribution of *x* |
| 7 | Count above mean | Number of values higher than the mean |
| 8 | Kurtosis | The kurtosis of *x* |
| 9 | Number peaks | Counts values larger than *n* neighbors on both sides |
| 10 | Count below mean | Number of values smaller than the mean. |

Reference

1 H. Zhao, Y. Zhang, L. Han, W. Qian, J. Wang, H. Wu, J. Li, Y. Dai, Z. Zhang, C. R. Bowen, Y. Yang, *Nano-Micro Letters* **2023**, *16* (1), 11, https://doi.org/10.1007/s40820-023-01216-0.

2 Z. Yue, X. Ye, S. Liu, Y. Zhu, H. Jiang, Z. Wan, Y. Lin, C. Jia, *Biosensors and Bioelectronics* **2019**, *139*, 111296, https://doi.org/https://doi.org/10.1016/j.bios.2019.05.001.

3 S. Wang, X. Fan, Z. Zhang, Z. Su, Y. Ding, H. Yang, X. Zhang, J. Wang, J. Zhang, P. Hu, *ACS Nano* **2024**, *18* (26), 17175, https://doi.org/10.1021/acsnano.4c04100.

4 S. Wang, F. Gao, Y. Hu, S. Zhang, H. Shang, C. Ge, B. Tan, X. Zhang, J. Zhang, P. Hu, *Chemical Engineering Journal* **2022**, *443*, 136446, https://doi.org/https://doi.org/10.1016/j.cej.2022.136446.

5 B. Yin, X. Liu, H. Gao, T. Fu, J. Yao, *Nature Communications* **2018**, *9* (1), 5161, https://doi.org/10.1038/s41467-018-07672-2.

6 Y. Yue, N. Liu, W. Liu, M. Li, Y. Ma, C. Luo, S. Wang, J. Rao, X. Hu, J. Su, Z. Zhang, Q. Huang, Y. Gao, *Nano Energy* **2018**, *50*, 79, https://doi.org/https://doi.org/10.1016/j.nanoen.2018.05.020.

7 K. Wang, Z. Lou, L. Wang, L. Zhao, S. Zhao, D. Wang, W. Han, K. Jiang, G. Shen, *ACS Nano* **2019**, *13* (8), 9139, https://doi.org/10.1021/acsnano.9b03454.

8 C.-B. Huang, S. Witomska, A. Aliprandi, M.-A. Stoeckel, M. Bonini, A. Ciesielski, P. Samorì, *Advanced Materials* **2019**, *31* (1), 1804600, https://doi.org/https://doi.org/10.1002/adma.201804600.

9 Y.-C. Huang, Y. Liu, C. Ma, H.-C. Cheng, Q. He, H. Wu, C. Wang, C.-Y. Lin, Y. Huang, X. Duan, *Nature Electronics* **2020**, *3* (1), 59, https://doi.org/10.1038/s41928-019-0356-5.

10 N. Bai, L. Wang, Q. Wang, J. Deng, Y. Wang, P. Lu, J. Huang, G. Li, Y. Zhang, J. Yang, K. Xie, X. Zhao, C. F. Guo, *Nature Communications* **2020**, *11* (1), 209, https://doi.org/10.1038/s41467-019-14054-9.
